# Characterization and fungicide sensitivity of *Trichoderma* species causing green mold of *Ganoderma sichuanense* in China

**DOI:** 10.3389/fmicb.2023.1264699

**Published:** 2023-10-19

**Authors:** Xuefei Li, Frederick Leo Sossah, Yonglan Tuo, Jiajun Hu, Qian Wei, Shiyu Li, Na Rong, Michael Wiafe-Kwagyan, Changtian Li, Bo Zhang, Xiao Li, Yu Li

**Affiliations:** ^1^Joint International Research Laboratory of Modern Agricultural Technology, Ministry of Education, Jilin Agricultural University, Changchun, China; ^2^Engineering Research Center of Chinese Ministry of Education for Edible and Medicinal Fungi, Jilin Agricultural University, Changchun, China; ^3^College of Plant Protection, Jilin Agricultural University, Changchun, China; ^4^Coconut Research Programme, Council for Scientific and Industrial Research (CSIR), Oil Palm Research Institute, Kade, Ghana; ^5^Department of Plant and Environmental Biology, School of Biological Sciences, College of Basic and Applied Sciences, University of Ghana, Accra, Ghana

**Keywords:** *Trichoderma* spp., *Ganoderma sichuansense*, green mold disease, pathogenicity, fungicides, prochloraz manganese, mushroom health

## Abstract

Green mold disease, caused by *Trichoderma* spp., is one of the most devastating diseases of mushrooms in China. The application of fungicides remains one of the important control methods among the integrated pest management tools for disease management in mushroom farms. This study aimed to identify *Trichoderma* spp., isolated from *G. sichuanense* fruiting bodies displaying green mold symptoms collected from mushroom farms in Zhejiang, Hubei, and Jilin Province, China, and evaluate their in vitro sensitivity to six fungicides. A total of 47 isolates were obtained and classified into nine *Trichoderma* spp. namely, *T*. *asperellum*, *T*. *citrinoviride*, *T*. *ganodermatiderum*, *T*. *guizhouense*, *T*. *hamatum*, *T*. *harzianum*, *T*. *koningiopsis*, *T*. *paratroviride*, and *T. virens*, through morphological characteristics and phylogenetic analysis of concatenated sequences of translation elongation factor 1-alpha (TEF) and DNA-dependent RNA polymerase II subunit (RPB2) genes. The pathogenicity test was repeated two times, and re-isolation of the nine *Trichoderma* spp. from the fruiting bodies of *G. sichuanense* fulfilled Koch’s postulates. Prochloraz manganese showed the best performance against most species. This research contributes to our understanding of green mold disease, reveals the phylogenetic relationships among *Trichoderma* species, and expands our knowledge of *Trichoderma* species diversity associated with green mold disease in *G. sichuanense*.

## Introduction

*Ganoderma sichuanense* is a widely distributed pore fungus that holds ecological and economic significance (Zhao et al., 1983; Wang et al., 2012). With its valuable medicinal properties, it has been cultivated for centuries in China, Japan, South Korea, and other regions (Zhu et al., 2019; Wang et al., 2020). In China, *G. sichuanense* has been cultivated for over 100 years, primarily in provinces such as Jilin, Heilongjiang, Shandong, Anhui, Guangdong, Guangxi, Fujian, Jiangxi, and Zhejiang. Recent studies have highlighted its medicinal benefits, including anti-tumor activity, antioxidant effects, blood sugar and lipid regulation, blood pressure reduction, antiviral activity, liver protection, and anti-aging effects (Xiao et al., 2016; Chiu et al., 2017; Rahman et al., 2018, 2020; Pan and Lin, 2019; Qiu et al., 2019; Wu et al., 2019; Krobthong et al., 2021).

The commercial expansion of *G. sichuanense* cultivation has become crucial due to limited wild germplasm resources. In 2020, China’s *Ganoderma* production exceeded 189,000 tons, representing significant economic value (China Edible Fungi Association, 2020). However, this expansion has also led to increased disease occurrences, resulting in substantial economic losses by impacting the quality and yield of *G. sichuanense*. Among the various fungal pathogens affecting *G. sichuanense* production, *Trichoderma* spp., *Xylogone ganodermophthora*, and *Cladobotryum* spp. pose significant challenges (Kang et al., 2010; Zuo et al., 2016; Yan et al., 2019; Cai et al., 2020).

Green mold disease, primarily caused by *Trichoderma* species, is particularly concerning as it hampers the growth and productivity of *G. sichuanense* (Wang et al., 2016). While *Trichoderma* is known for its biocontrol effects, it can also act as a pathogen, posing a serious threat to edible fungi during cultivation (Shah et al., 2013; Kosanović et al., 2020). The occurrence of green mold disease caused by *Trichoderma* species in China has raised significant concerns, resulting in contamination and losses in yield and quality (Seaby, 1987; Choi et al., 1998; Wang et al., 2016; Seung et al., 2018). The impact of this disease on *G. sichuanense* cultivation in China is of particular concern given the economic importance of this valuable medicinal fungus (Pan and Lin, 2019; Rahman et al., 2020).

In the context of *Ganoderma* cultivation, *Trichoderma*-induced diseases are particularly problematic during the mycelial growth and emergence stages of *G. sichuanense*. However, limited research has been conducted on the diversity and pathogenicity of *Trichoderma* species isolated from *G. sichuanense* in China, and the establishment of effective control measures against *Ganoderma*-related diseases remains a challenge (Seaby, 1987; Chen and Zhuang, 2017; Yan et al., 2019; Cai et al., 2020; An et al., 2022). Therefore, identifying the causal agent and understanding its pathogenicity are crucial prerequisites for the development of effective disease management strategies.

Although fungicides are effective in controlling green mold disease, their use can lead to the development of resistance and pose environmental risks. Nevertheless, fungicides remain the most effective measure for disease control (Shah et al., 2013; Kosanović et al., 2015; Innocenti et al., 2019). Understanding the sensitivity of *Trichoderma* species to various fungicides can significantly contribute to disease management strategies. However, the fungicide sensitivities of *Trichoderma* isolates causing green mold disease in *G. sichuanense* in China have not been thoroughly investigated.

In this study, we aimed to investigate the *Trichoderma* species associated with *G*. *sichuanense* and their impact on disease development. Our findings revealed a disease incidence ranging from 3 to 15%, which significantly affected the growth and development of *G*. *sichuanense*, leading to direct economic consequences. The rapid germination and spread of *Trichoderma* spores underscored the potential for irreparable damage if the disease is not promptly controlled. We focused on the identification and characterization of these *Trichoderma* species, the assessment of their pathogenicity in *G*. *sichuanense*, and the evaluation of their sensitivity to fungicides. Through these comprehensive analyses, our objective was to provide valuable insights into disease management strategies for *G*. *sichuanense* cultivation.

## Materials and methods

### Sample collection and fungal isolation

During the period from 2021 to 2022, we collected fruiting bodies (basidiomata) of *G. sichuanense* displaying symptoms of green mold disease from three farms situated in Zhejiang, Hubei, and Jilin Province, China. The incidence of the disease ranged from 3 to 15%, significantly impacting the growth and development of *G. sichuanense*.

To conduct a comprehensive investigation of the disease, we isolated the fungus from the infected fruiting bodies using the tissue-isolation method. This involved carefully excising small pieces (0.3 cm) from the edges of the lesions on the diseased fruiting bodies using a sterile scalpel. The excised tissues were then subjected to surface sterilization by treating them with 75% ethanol (vol/vol) for 30 s, followed by 1% NaOCl (wt/vol) for 10 s. Subsequently, the tissues underwent three rinses with sterilized distilled water.

The tissues were placed onto dried and sterilized potato dextrose agar (PDA) plates and incubated in darkness at 25°C for three to 5 days. Regular inspections were carried out to monitor any fungal growth. Colonies that developed from the infected tissues were transferred to new PDA plates using the hyphal tip culture method to obtain pure cultures. All purified isolates were further subcultured on PDA medium for 3 days and preserved on PDA slants at 4°C.

### Morphological characterization

To evaluate the characteristics of the isolates, mycelia plug with a diameter of 5.0 mm were obtained from the edges of actively growing cultures aged 5 days. These plugs were then placed at the center of agar plates containing potato dextrose agar (PDA), cornmeal dextrose agar (CMD), and synthetic low-nutrient agar (SNA). The plates were incubated at 25°C with a 12-h light/dark photoperiod for a duration of 5–7 days.

During the incubation period, careful observations and recordings were made on various colony characteristics, including color, shape, radial growth, and texture. The colony diameters were measured in two perpendicular directions. The daily growth rate was determined by calculating the average mean daily growth (mm/day).

For further analysis, one-week-old colonies cultivated on SNA plates were utilized to examine the conidia and conidiophores following the methods outlined by Chaverri et al. (2015). The shape and color of the conidia were observed, and the sizes of 20 randomly selected conidia from each isolate were measured under a Zeiss Axio lab. A1 microscope equipped with a differential interference contrast (DIC) optics camera (Carl Zeiss Microscopy GmbH, Germany), utilizing 1,000× magnification.

### DNA extraction and sequence analysis

To obtain DNA for analysis, mycelia were collected from colonies cultivated on potato dextrose agar (PDA) for 3–5 days. DNA extraction was performed using the NuClean Plant Genomic DNA Kit (Cowin Biotech Co., Ltd., Taizhou, China).

For amplification of the target genes, specific primer pairs were used. The primer pair fRPB2-5f and fRPB2-7cr (Liu et al., 1999) amplified a 1 kb fragment of the RNA polymerase II second largest subunit (RPB2) gene. Additionally, the primer pair EF1-728F and TEF1LLErev (Chaverri et al., 2003; Jaklitsch et al., 2005) amplified a 1.3 kb fragment of the translation elongation factor 1-alpha (TEF1-a) gene. PCR amplification was conducted in a 30 μL reaction system comprising 15 μL of 10× PCR mix, 1.5 μL of each primer, 1.5 μL of template DNA, and 10.5 μL of ddH_2_O. For both RPB2 and TEF1-a genes, PCR conditions included an initial denaturation step at 95°C for 5 min, followed by 30 cycles of denaturation at 95°C for 1 min, annealing at 59°C for RPB2 or 55°C for TEF1-a for 90 s, extension at 72°C for 90 s, and a final extension at 72°C for 10 min. The PCR products were purified using the PCR Product Purification Kit, and gel electrophoresis was performed to confirm successful amplification.

Sequencing of the PCR products was carried out bidirectionally using the fRPB2-5f/fRPB2-7cr and TEF1/TEF2 primers (Jaklitsch, 2009) at Comate Biosciences Co. Ltd (Changchun, Jilin, China). The obtained sequences were assembled using CAP3 software (Huang and Madan, 1999) to generate consensus sequences. BioEdit software (version 7.0.0) was used to remove 20 to 30 bp from the terminal ends. Basic Local Alignment Search Tool (BLAST) analysis^1^ was conducted for each gene locus to confirm the identity of the isolates. The consensus sequences were deposited in GenBank (Table 1).^2^

**Table 1:** Specimen Numbers, country and their corresponding GenBank accession numbers of sequences used for phylogenetic analyses.

Scientific name	Specimen numbers	Country	Substrate	GenBank accession numbers		
				RPB2	TEF1-a	ITS
*T. anisohamatum*	YMF1.00333 T	China	/	MH155272	MH177912	MH113926
*T. anisohamatum*	YMF1.00215	China	/	MH262576	MH236494	MH262583
*T. asperellum*	CBS 433.97 T	USA	Soil	EU248617	AY376058	/
**T. asperellum**	**T19**	**China**	**G. sichuanense**	**OR291404**	**OR291385**	**OR569146**
*T. atroviride*	CBS 142.95 ET	Slovenia	Decayed log	EU341801	AF456891	MH862505
*T. atroviride*	NECC21247	/	/	OL790433	OL790432	OL690567
*T. ceramicum*	CBS 114576 T	USA	Wood	FJ860531	FJ860628	FJ860743
*T. ceramicum*	GJS 88–70 T	USA	Wood	AF545510	AF534593	AY737764
*T. citrinoviride*	DAOM 172792 T	/	/	KJ842210	KJ713208	EU280098
*T. citrinoviride*	DEMf:TR4	Serbia	*Pinus sylvestris* bark	OK422202	OK422205	OK384603
**T. citrinoviride**	**T31**	**China**	**G. sichuanense**	**OR291411**	**OR291392**	**OR569153**
*T. estonicum*	GJS 96–129 T	Estonia	*Hymenochaete tabacina*	AF545514	AF534604	AY737767
*T. ganodermatiderum*	CCMJ5245 T	China	*G. sichuanense*	ON567189	ON567195	ON399102
*T. ganodermatiderum*	CCMJ5246	China	*G. sichuanense*	ON567190	ON567196	ON399103
**T. ganodermatiderum**	**T1**	**China**	**G. sichuanense**	**OR291399**	**OR291380**	**OR569141**
**T. ganodermatiderum**	**T2**	**China**	**G. sichuanense**	**OR291400**	**OR291381**	**OR569142**
**T. ganodermatiderum**	**T3**	**China**	**G. sichuanense**	**OR291401**	**OR291382**	**OR569143**
*T. guizhouense*	HGUP0038 T	China	Soil	JQ901400	JN215484	JN191311
*T. guizhouense*	S278	Croatia	/	KF134791	KF134799	/
**T. guizhouense**	**T41**	**China**	**G. sichuanense**	**OR291413**	**OR291394**	**OR569155**
**T. guizhouense**	**T42**	**China**	**G. sichuanense**	**OR291414**	**OR291395**	**OR569156**
*T. hamatum*	DAOM 167057 ET	Canada	/	AF545548	EU279965	EU280124
*T. hamatum*	KUFA 0088	/	/	OP250964	OP250957	OP218247
**T. hamatum**	**T28**	**China**	**G. sichuanense**	**OR291410**	**OR291391**	**OR569152**
*T. harzianum*	CBS 226.95 T	England	Soil	AF545549	AF348101	AJ222720
*T. harzianum*	GJS 05–107	Italy	*Ricinus communis*	FJ442708	FJ463329	/
**T. harzianum**	**T23**	**China**	**G. sichuanense**	**OR291407**	**OR291388**	**OR569149**
**T. harzianum**	**T24**	**China**	**G. sichuanense**	**OR291408**	**OR291389**	**OR569150**
*T. koningiopsis*	GJS 93–20 T	Cuba	Branch	EU241506	DQ284966	DQ313140
*T. koningiopsis*	CCMJ5254	China	*G. sichuanense*	ON567202	ON567188	ON385947
**T. koningiopsis**	**T26**	**China**	**G. sichuanense**	**OR291409**	**OR291390**	**OR569151**
**T. koningiopsis**	**T40**	**China**	**G. sichuanense**	**OR291412**	**OR291393**	**OR569154**
**T. koningiopsis**	**T43**	**China**	**G. sichuanense**	**OR291415**	**OR291396**	**OR569157**
**T. koningiopsis**	**T45**	**China**	**G. sichuanense**	**OR291416**	**OR291397**	**OR569158**
*T. paratroviride*	S385 T	Spain	/	KJ665321	KJ665627	/
*T. paratroviride*	PARC1012	/	/	MT454131	MT454115	MT448958
**T. paratroviride**	**T17**	**China**	**G. sichuanense**	**OR291402**	**OR291383**	**OR569144**
**T. paratroviride**	**T18**	**China**	**G. sichuanense**	**OR291403**	**OR291384**	**OR569145**
**T. paratroviride**	**T47**	**China**	**G. sichuanense**	**OR291417**	**OR291398**	**OR569159**
*T. parestonicum*	CBS 120636 T	Austria	*Hymenochaete tabacina*	FJ860565	FJ860667	FJ860803
*T. virens*	DIS 162	Costa Rica	*T. cacao*	FJ442696	FJ463367	FJ442669
*T. virens*	DIS 328A	Ecuador	*T. gileri*	FJ442738	FJ463363	FJ442670
**T. virens**	**T20**	**China**	**G. sichuanense**	**OR291405**	**OR291386**	**OR569147**
**T. virens**	**T21**	**China**	**G. sichuanense**	**OR291406**	**OR291387**	**OR569148**

Sequences produced in this study are in bold.

### Phylogenetic analyses

After performing a BLAST search using the obtained ITS, RPB2, and TEF1-a sequences in the NCBI GenBank database, sequences that met specific criteria: ≥ 99% similarity for RPB2, ≥ 97% for TEF1-a, and ≥ 76% for ITS, were utilized to verify the identity of Trichoderma species in our phylogenetic analysis (Cai and Druzhinina, 2021). We retrieved homologous RPB2, TEF1-a, and ITS gene sequences of the isolates from GenBank. These sequences were aligned using the MUSCLE program (Edgar, 2004), and the resulting alignment was further refined using BioEdit 7.2.5 (Hall, 1999; Hall, 2011). Finally, we concatenated the gene sequences using Phylosuit V1.2.2 (Zhang et al., 2020). For the phylogenetic analysis, we employed the Maximum-Likelihood (ML) method using PhyML 3.0 (Guindon et al., 2010). The best substitution model was determined with PartitionFinder v2.1.1 (Lanfear et al., 2017). To assess statistical support, we conducted bootstrapping with 1,000 replicates (ML). Detailed lists of the fungal isolates used in this study can be found in Table 1 and Supplementary Table S1. The resulting ML tree was visualized using Figtree v1.4.4,^3^ providing a clear representation of the phylogenetic relationships among the isolates.

### Pathogenicity tests

Pathogenicity experiments were conducted following Koch’s postulates, with each experiment replicated twice to ensure accuracy. Fully colonized substrate bags containing *G*. *sichuanense* were sourced from the Panshi Mushroom Base in Jilin Province, China. These bags were placed in a growth room with controlled conditions, including a temperature range of 25–30°C and humidity levels set between 80 and 90%, to promote fruiting. Once the fruiting bodies were formed, the bottom surface of the cap and the stipe were meticulously damaged using a sterilized needle. Subsequently, they were inoculated with a spore suspension of the isolates at a concentration of 1 × 10^5^ spores per milliliter. As a comparison, the control group was inoculated with sterilized distilled water.

For each strain, six bags of *G*. *sichuanense* were inoculated. The development of symptoms was monitored daily for a period of 14 days. To confirm the causative agents of green mold disease, the pathogens were re-isolated from the inoculated *G*. *sichuanense* showing green mold symptoms. Identification was performed using the aforementioned morphological and molecular methods, considering strains that matched the original inoculum as the causative agents of green mold disease.

### Effect of *Trichoderma* spp. on *G. sichuanense* mycelia in petri plates

To assess the aggressiveness of the isolates, a subset of nine isolates representing nine different species was selected from the total of 47 isolates. The experiments were performed with three replicates, following the procedure outlined below. Mycelial agar plugs with a diameter of 8 mm were obtained from the advancing edge of 10-day-old *G. sichuanense* colonies. These plugs were then inoculated onto potato dextrose agar (PDA) plates, positioned 1 cm from the edge of Petri plates with a diameter of 9 cm. After 7 days, mycelial plugs from *Trichoderma* cultures were inoculated in the same manner, but on the opposite side of the plate, 1 cm away from the edge. The growth of *Trichoderma* species in confrontation with *G. sichuanense* mycelia was carefully observed and recorded.

### Fungicide sensitivity of isolates and *G. sichuanense*

To evaluate the efficacy of fungicides against green mold in mushrooms, a preliminary screening of six fungicides (mancozeb, chlorothalonil, fludioxonil, carbendazim, prochloraz, and prochloraz-Mn) was conducted. Stock solutions of each fungicide at a concentration of 100 mg/mL were prepared by dissolving them in sterilized distilled water. The growth inhibition rate of the fungi was assessed through mycelial growth assays.

PDA medium plates with different concentrations of each fungicide were prepared by adding the appropriate volume of the stock solution to sterilized distilled water. Mycelial plugs with a diameter of 7 mm were obtained from the edges of 3-day-old colonies grown on PDA and placed at the center of the PDA plates containing varying fungicide concentrations. All plates were then incubated at 25°C for 3 days. The growth inhibition rate of the mycelia was calculated using the formula i=a1−a2a1×100 , where “*i*” represents the growth inhibition rate, “a1” is the hyphae area of the untreated pathogen, and “a2” is the hyphae area of the treated pathogen (Etebarian et al., 2005).

Each fungicide treatment and the control were replicated on three plates, and the experiment was repeated twice. Based on the preliminary screening results of the six fungicides using the nine isolates, a suitable fungicide was selected. The sensitivity of *G. sichuanense* to these fungicides was further tested using the same method described above.

The sensitivity of the fungi to fungicides was determined by measuring the fungicide concentration that inhibited fungal development by 50% [half maximal effective concentration (EC50)] (Wong and Midland, 2007; Kim et al., 2020). The relative growth (RG) of the fungi at a specific fungicide concentration was calculated as a percentage of fungal growth compared to the control plates. The EC50 value was obtained by performing linear regression analysis on the probit-transformed relative inhibition values (1 - RG) at log10-transformed fungicide concentrations. The EC50 value for each isolate was calculated as the average of three experiments. The correlation coefficients (*r*) among EC50 values for different fungicides were determined using statistical algorithms provided by SAS software (version 9.4 for Windows; SAS Institute, Cary, NC, U.S.A.).

## Results

### Disease symptoms and fungal isolation

**Figure 1:**
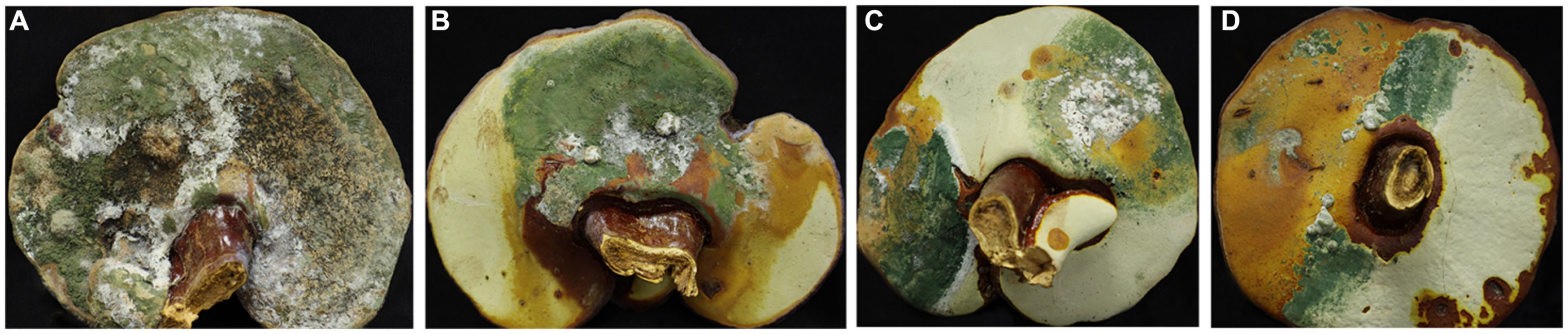
*Ganoderma sichuanense* fruiting bodies infected by *Trichoderma* **(A–D)**.

The fruiting bodies of *G. sichuanense* exhibited symptoms of green mold disease, which were visually distinct. Infected basidiomata displayed a layer of green mycelia, leading to decay and withering of the affected fruiting bodies (Figure 1). The severity of the disease was evident, as it progressed rapidly, particularly after watering flushes of the fruiting bodies. The development of symptoms followed a specific pattern: initially, white spots and mycelium appeared on the infected fruiting bodies. Under hot weather conditions or high humidity, there was a significant proliferation of green conidia within a short time, gradually covering the entire surface of the fruiting bodies. Subsequently, the spores dispersed through various means, such as water flow, human movement, or wind, resulting in the demise of *G. sichuanense* fruiting bodies and the loss of their ability to produce spores.

To investigate the pathogens responsible for green mold disease, we conducted fungal isolation from the infected fruiting bodies. A total of 47 pathogens were isolated and identified during the study (Tables 1 2; Supplementary Table S1). Among the isolated pathogens, we identified one strain of *T*. *harzianum* in Zhejiang Province and three strains in Hubei Province, namely *T*. *koningiopsis*, *T*. *paratrovide*, and *T. virens*. Interestingly, in Jilin Province, we observed a diverse range of strains, with a total of nine different species identified (Table 2). These pathogens were characterized by their ability to induce the distinctive symptoms associated with green mold disease on *G*. *sichuanense*.

**Table 2:** Number of *Trichoderma* isolates recovered from *G. sichuanense* with macroscopic symptoms of green mold disease collected.

Species	*T. ganodermatiderum*	*T. koningiopsis*	*T. paratroviride*	*T. harzianum*	*T. virens*	*T. guizhouense*	*T. hamatum*	*T. asperellum*	*T. citrinoviride*
Strains	T1-T16, T30, T32, T36, T37, T38, T46, T48	T26, T27, T33, T39, T40, T43, T44, T45	T17, T18, T34, T35, T47	T23, T24, T25	T20, T21, T22	T41, T42	T28	T19	T31
Total	23	8	5	3	3	2	1	1	1
^a^Percentage	48.93%	17.02%	10.64%	6.38%	6.38%	4.26%	2.13%	2.13%	2.13%

a Percentage = *n*/*N* × 100%, where *n* is the number of isolates for one species of Trichoderma, and *N* is the total number of isolates for all Trichoderma species.

### Morphological characteristics

**Figure 2:**
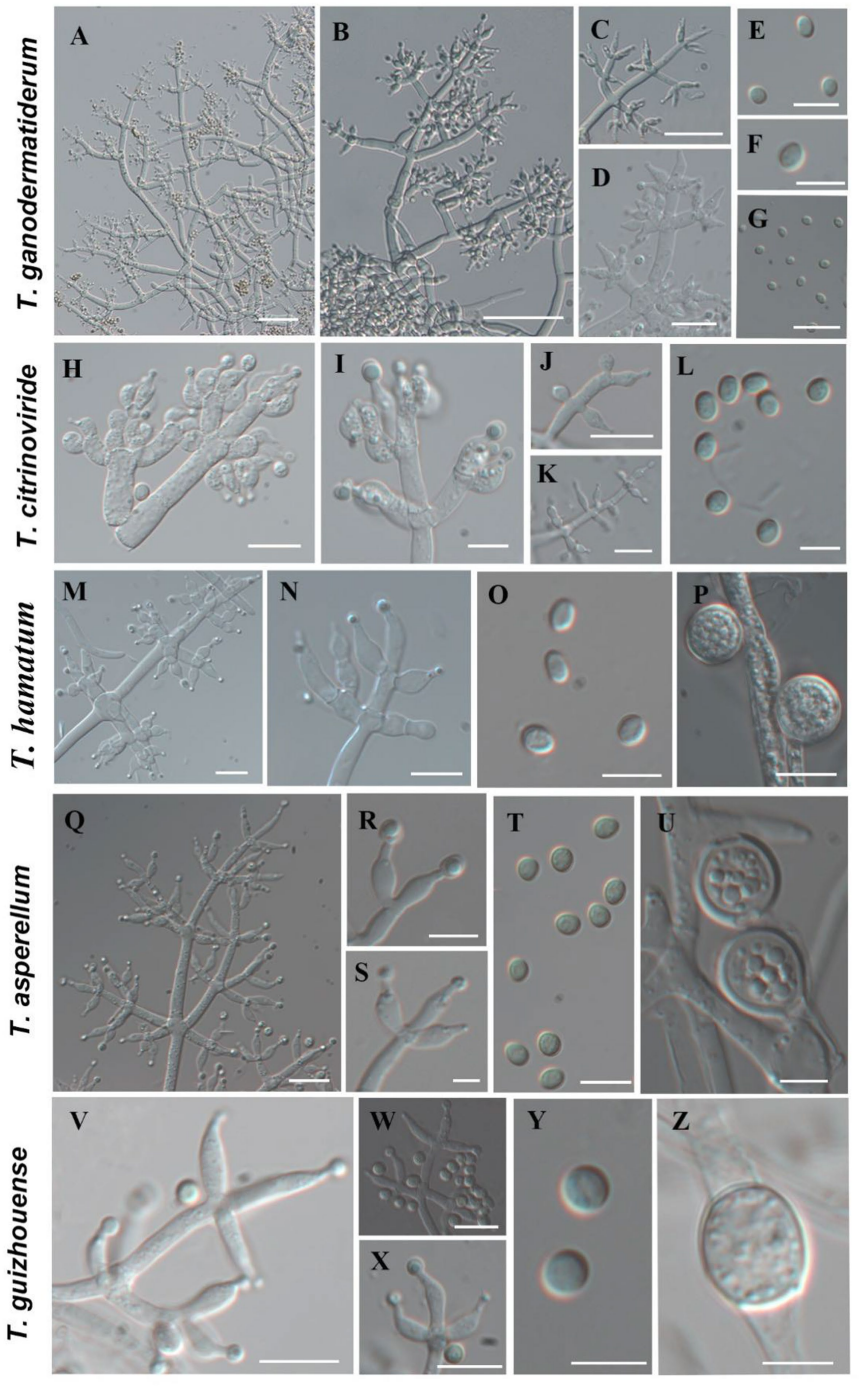
Morphological characteristics of *T. ganodermatiderum*, *T. citrinoviride*, *T. hamatum*, *T. asperellum*, *T. guizhouense*. (Scale bars: **A–D**, **J** = 40 μm; **G,K,M,N,Q** = 20 μm; **E,F,H,I,O,P,R–T,V–X** = 10 μm; **L,U,Y,Z** = 5 μm).

**Figure 3:**
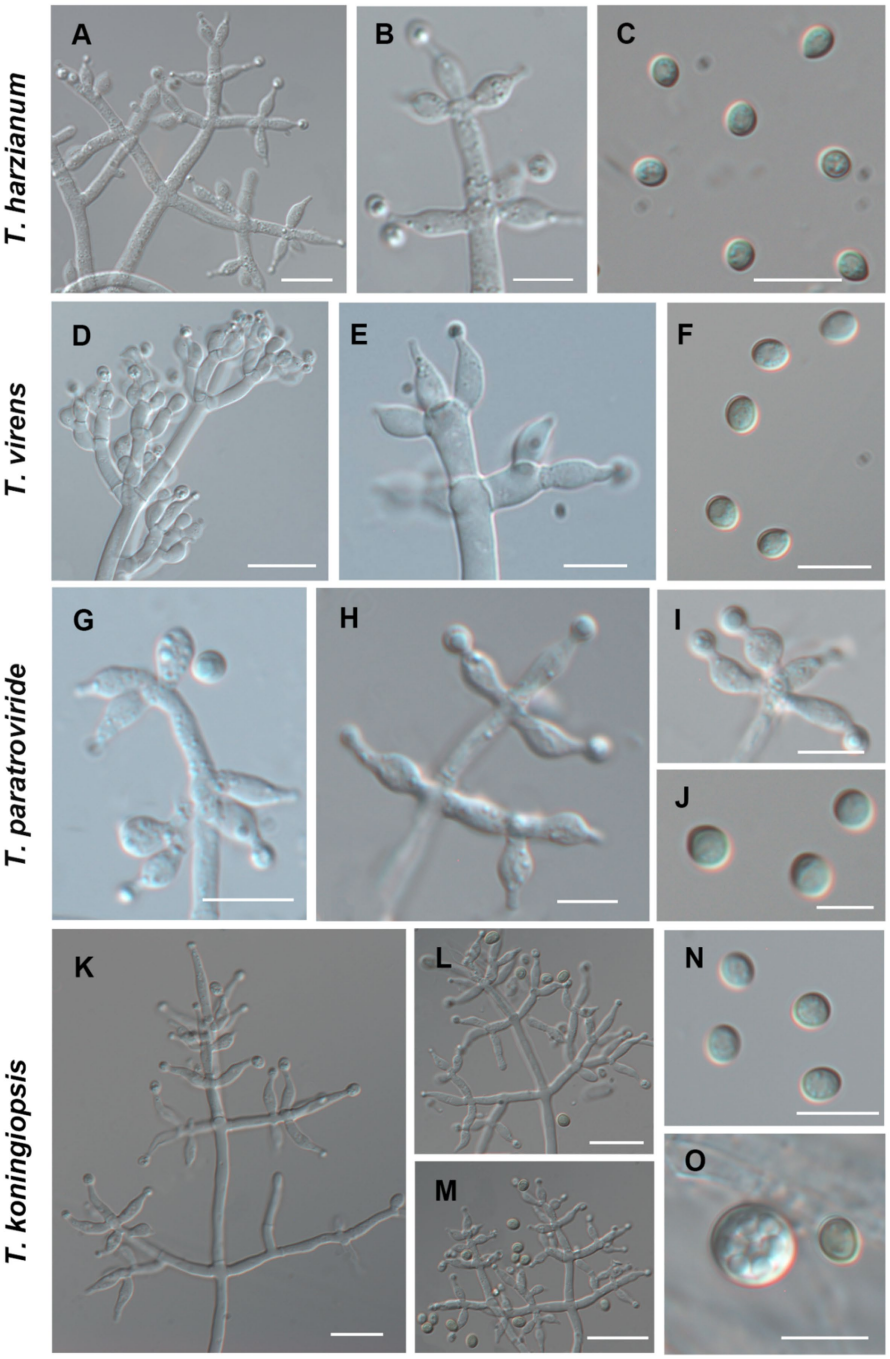
Morphological characteristics of *T. harzianum, T. virens, T. paratroviride, T. koningiopsis.* (Scale bars: **A,D,E,K–M** = 40 μm; **B,C,F,G–I,N,O** = 10 μm; **J** = 5 μm).

Using the classification methods proposed by Bissett (1984), Gams and Bissett (1998), and Park et al. (2006), we conducted a meticulous examination of colony shape, conidia, conidiophore size, chlamydospores, and pigmentation (Figures 2, 3) to identify nine *Trichoderma* species. The isolated species include *T*. *ganodermatiderum* (Figures 2A–G), *T*. *citrinoviride* (Figures 2H–L), *T*. *hamatum* (Figures 2M–P), *T*. *asperellum* (Figures 2Q–U), *T*. *guizhouense* (Figures 2V–Z), *T*. *harzianum* (Figures 3A–C), *T. virens* (Figures 3D–F), *T*. *paratroviride* (Figures 3G–J), and *T*. *koningiopsis* (Figures 3K–O).

**Figure 4:**
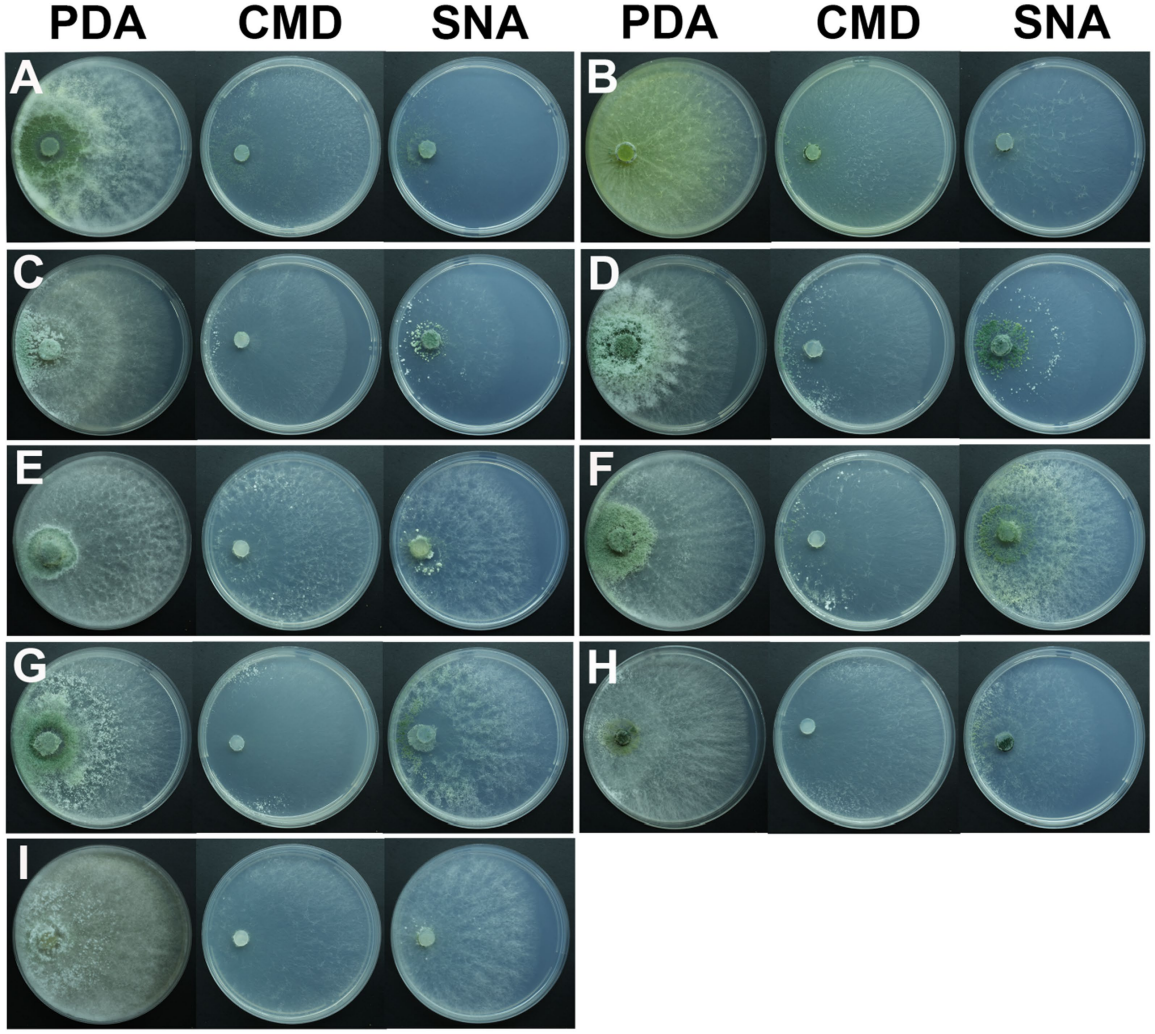
Colony appearance of representative isolates of 9 *Trichoderma* species. **(A)** *T. ganodermatiderum*; **(B)** *T. citrinoviride*; **(C)** *T. hamatum*; **(D)** *T. asperellum*; **(E)** *T. guizhouense*; **(F)** *T. harzianum*; **(G)** *T. virens*; **(H)** *T. paratroviride*; **(I)** *T. koningiopsis*.

After 1 day of cultivation at 25°C, all strains displayed white villous colonies on PDA, SNA, and CMD media. By the fifth day, light green to dark green sporulation bands emerged on all media, gradually extending toward the center. CMD and SNA media supported the growth of relatively thin colonies. By the seventh day, green spores were dispersed throughout the entire plate, exhibiting a grayish green or chartreuse color (Figure 4). *T. citrinoviride* exhibited the production of a yellow pigment at a later stage (Figure 4B), and some exhibited concentric rings (Figure 4).

Microscopic analysis unveiled notable distinctions in the morphology of conidiophores, phialides, and conidia among the *Trichoderma* species. *T. ganodermatedrum* (Figures 2A–G), *T. asperellum* (Figures 2Q–U), *T. harzianum* (Figures 3A–C), and *T. koningiopsis* (Figures 3K–O) exhibited dendriform branches and green spherical or ellipsoidal spores. While *T. ganodermatedrum* (Figures 2A–G) and *T. harzianum* (Figures 3A–C) shared similar spore sizes, the former displayed densely distributed conidiophores, whereas the latter had sparser conidiophore clusters. *T. asperellum* (Figures 2Q–U) and *T. koningiopsis* (Figures 3K–O) exhibited similar spore sizes, but there were significant differences in the sizes of their phialides. Specifically, *T. koningiopsis* had phialides measuring 5.0–7.5 × 3.0–4.8 μm (Figures 3K–M), while *T. asperellum* had phialides measuring 7–11 × 2–4 μm (Figures 2Q–S).

*T. virens* presented irregular branches at the top of its conidiophores, often accompanied by 3–6 closely arranged phialides, resulting in a more complex structure (Figures 3D–F). *T. hamatum* displayed highly branched conidiophores, primarily with opposite lateral branches and a few solitary branches (Figures 2M–P). The phialides of *T. hamatum* were densely packed, short, and round, measuring 5–7.5 × 3.0–4.4 μm (Figures 2M–N). In the case of *T. citrinoviride*, its conidiophores appeared either opposite or alternate, and it possessed small spores measuring 2.9–4.0 × 1.8–2.2 μm (Figures 2H–L). While *T. guizouense* (Figures 2V–Z) and *T. paratroviride* (Figures 3G–J) featured nearly spherical spores, the former exhibited conidiophores in pairs or whorls, with phialides typically arranged in groups of 2–4. Conversely, *T. guizouense* predominantly displayed conidiophores in 2–3 whorls, occasionally occurring solitary, and its phialides were symmetrically distributed (Figures 2V–X). Notably, *T. hamatum* (Figure 2P), *T. asperellum* (Figure 2U), *T. guizouense* (Figure 2Z), and *T. koningiopsis* (Figure 3O) exhibited abundant chlamydospores in later stages. For further details regarding the specific characteristics of each *Trichoderma* isolate, please consult Table 3.

**Table 3:** Microscopic characteristics of different *Trichoderma* isolates.

Species	Conidiophores and phialides	Conidia	Chlamydospores
*T. ganodermatiderum*	Tree-like, straight or slightly curved, with visible main axis and densely distributed branches. Phialides arranged in pairs or 3–5 wheels, 2.5–10.0 × 2.2–3.5 μm (Figures 2A–D)	Green, smooth-walled, subglobose to ellipsoidal, 3.0–4.8 × (2.5-) 2.8–3.8 μm (Figures 2E–G)	Not found
*T. citrinoviride*	Opposite or alternate, tree-like, with long main axis and short secondary branches. Phialides with 2–3 in 1 round, 3.5–5.2 × 1.8–3.5 μm (Figures 2H–K)	Chartreuse to green, smooth, ellipsoidal, 2.9–4.0 × 1.8–2.2 in size μm (Figure 2L)	Not found
*T. hamatum*	Main axis straight and highly branched, lateral branches opposite, few solitary. Phialides dense, short and chubby, 5–7.5 × 3.0–4.4 μm (Figures 2M,N)	Light green, smooth-walled, oblong, 4.1–5.0 × (2.5-) 3.0–3.5 μm (Figure 2O)	Spherical, terminal and intercalary, 8–12 × 6–10 μm (Figure 2P)
*T. asperellum*	Tree-like, lateral branches opposite, nearly perpendicular with main axios. Phialides symmetrically distributed, 7–11 × 2–4 μm (Figures 2Q–S)	Ellipsoidal, 3.5–5.0 × 3.0–4.2 μm (Figure 2T)	Subglobose, terminal or occasionally interstitial, smooth, 7–10 μm (Figure 2U)
*T. guizhouense*	Conidiophores in 2–3 whorls, occasional solitary growth. Phialides symmetrically distributed, conical, with a thin top, 5.5–11 × 2–3.5 μm (Figures 2V–X)	Spherical or subglobose, green, 2.0–3.2 × 2.0–3.0 μm (Figure 2Y)	Subglobose to ellipsoidal, intermediate, 5.5–8.6 × 4.7–7 μm (Figure 2Z)
*T. harzianum*	Tree-like, resembling a pyramid, main axis straight, many secondary branches. Phialides short, arranged in a circular pattern, usually in 3–4 whorls, occasionally opposite. (Figures 3A,B)	Spherical, subglobose, or obovate, smooth-walled, light green, 2.5–3.9 (−4.0) × 2.5–3.5 μm (Figure 3C)	Not found
*T. virens*	Irregular branching at the top, complex, with no branching at the base. Middle expansion of phialides, 4.0–6.5 × 3.0–5.0 μm (Figures 3D,E)	Green, smooth, broadly ellipsoid to obovate, 3.5–5.0 × 2.8–4.0 μm (Figure 3F)	Not found
*T. paratroviride*	Main axis is long, with branches in pairs or whorls, phialides usually arranged in 2–4 rounds, 5.0–8.5 × 2.5–3.0 μm (Figures 3G–I)	Subglobose, green, smooth, 3.0–4.0 × 3.0–3.5 μm (Figure 3J)	Not found
*T. koningiopsis*	Tree-like, longer main axis, branches growing alone or in pairs, at right angles to the main axis. Phialides slender, middle enlarged, 5.0–7.5 × 3.0–4.8 μm (Figures 3K–M)	Ellipsoidal, green, 4.0–5.0 × 2.8–3.2 μm (Figure 3N)	Spherical, green, 7.5–10.4 μm (Figure 3O)

### Phylogenetic analysis

**Figure 5:**
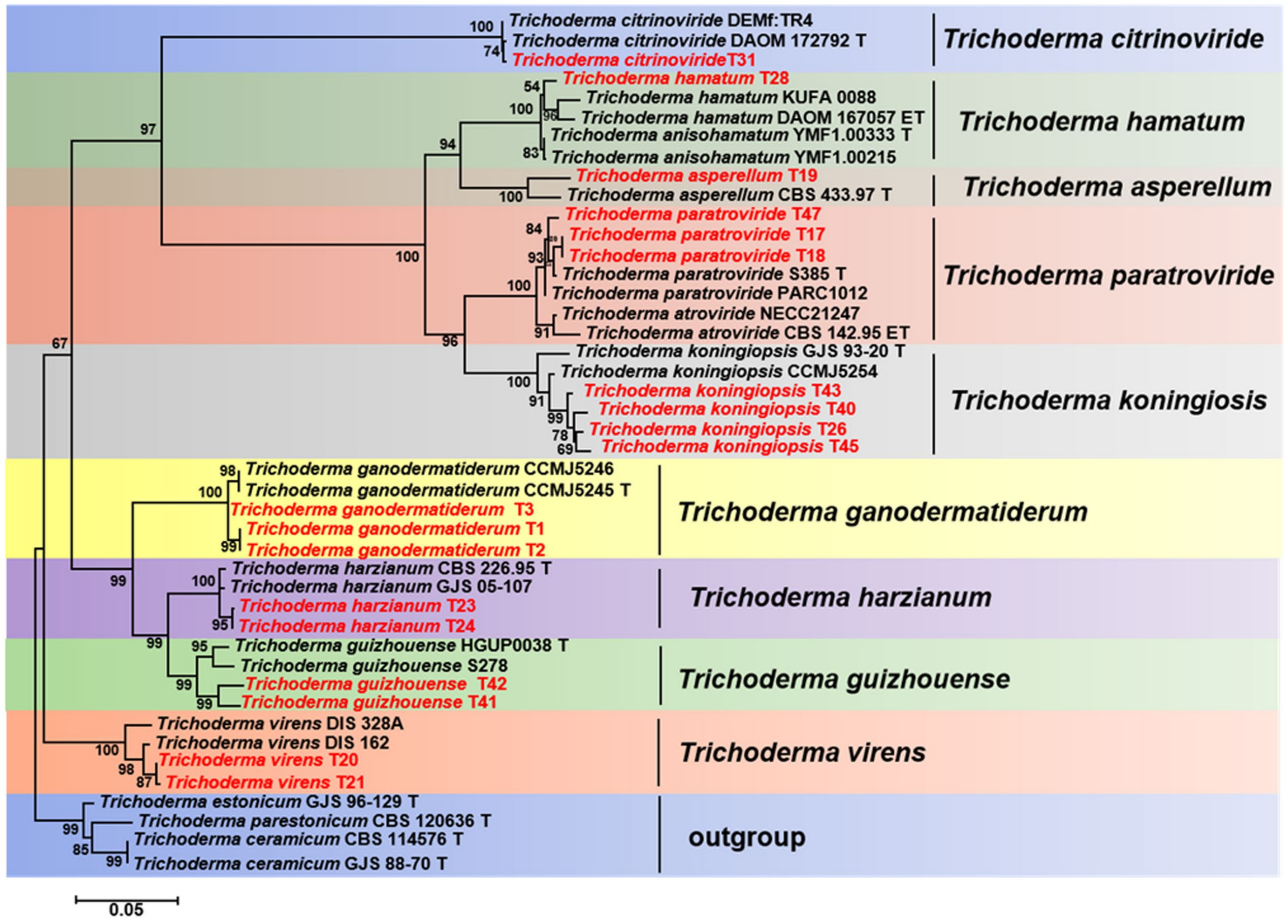
Phylogenetic tree illustrating the relationships among 19 *Trichoderma* isolates from *Ganoderma sichuanense* based on the combined TEF-1a and RPB2 genes using PhyML analysis. Bootstrap support values equal to or greater than 70% are shown at the nodes. *T. estonicum* and *T. ceramicum* were used as the outgroup. The isolates obtained in this study are highlighted in red.

The TEF1-a and RPB2 gene sequences of all *Trichoderma* isolates were compared to the NCBI database using BLAST analysis. Matches exhibiting a high similarity level (≥90%) were chosen for subsequent analysis. A phylogenetic analysis was conducted using the concatenated sequences of the TEF1-a and RPB2 genes from all *Trichoderma* isolates. The analysis revealed that the *Trichoderma* isolates could be classified into nine distinct clades: *T*. *asperellum*, *T*. *citrinoviride*, *T*. *ganodermatiderum*, *T*. *guizhouense*, *T*. *hamatum*, *T*. *harzianum*, *T*. *koningiopsis*, *T*. *paratroviride*, and *T. virens* (Figure 5). The phylogenetic trees were constructed using a dataset consisting of 19 sequences derived from two gene loci (TEF1-a and RPB2) obtained from a total of 47 samples. Among these sequences, 38 were newly generated, including 19 TEF1-a sequences and 19 RPB2 sequences. For more detailed information on the specific characteristics of each *Trichoderma* isolate, please refer to Table 1 and Supplementary Table S1.

### Pathogenicity tests

**Figure 6:**
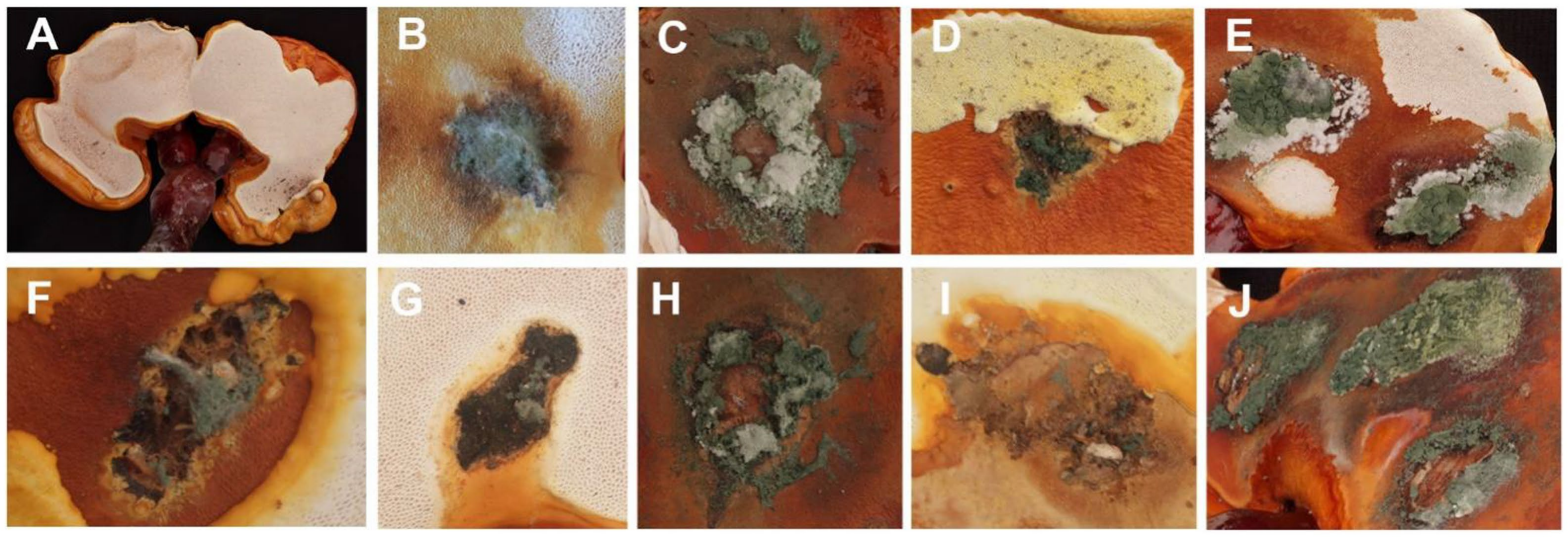
**(A)** CK; **(B)** *T. ganodermatiderum*; **(C)** *T. citrinoviride*; **(D)** *T. asperellum*; **(E)** *T. guizhouense*; **(F)** *T. hamatum*; **(G)** *T. virens*; **(H)** *T. paratroviride*; **(I)** *T. Koningiopsis*; **(J)** *T. harzianum.*

In the pathogenicity test, mechanical damage was induced on the fruiting bodies followed by *in vitro* inoculation using a spore suspension. Two weeks after inoculation, all *Trichoderma* species showed similar green mold symptoms, as observed in Figure 6. Initially, small oval spots with white to pale green centers surrounded by a chlorotic area appeared on the *G. sichuanense* fruiting bodies 7 days post-inoculation. Over time, these lesions progressively increased in size and merged together. In severe cases, the infected fruiting bodies were completely covered by green spores. These symptoms observed under greenhouse conditions were consistent with the field symptoms of *G. sichuanense*. No symptoms were observed in the control group (Figure 6A).

Additionally, all *Trichoderma* species were consistently re-isolated and confirmed using morphological and molecular methods, while no *Trichoderma* isolates were obtained from the control group, satisfying Koch’s postulates. The pathogenicity study demonstrated that all *Trichoderma* isolates induced green mold disease in *G. sichuanense* fruiting bodies upon inoculation. Among the isolates, *T. harzianum* exhibited the highest virulence (Figure 6J), followed by *T. citrinoviride* (Figure 6C), *T. paratroviride* (Figure 6H), *T. guizhouense* (Figure 6E), *T. ganodermatiderum* (Figure 6B), *T. asperellum* (Figure 6D), *T. virens* (Figure 6G), *T. koningiopsis* (Figure 6I), and *T. hamatum* (Figure 6F).

### Effect of *Trichoderma* spp. on *G. sichuanense* mycelia

To assess the impact of different *Trichoderma* species on *G. sichuanense* mycelia, we conducted plate dual culture experiments. The results revealed that all *Trichoderma* species inhibited the growth of *G. sichuanense* mycelia and produced antagonistic lines. However, there were variations in the interactions between the nine *Trichoderma* species and *G. sichuanense* mycelia (Supplementary Figure S2). Notably, *T. ganodermatiderum* (Supplementary Figure S2A), *T. citrinoviride* (Supplementary Figure S2B), *T. asperellum* (Supplementary Figure S2D), and *T. paratroviride* (Supplementary Figure S2H) exhibited significant inhibition on *G. sichuanense* mycelial growth, while *T. hamatum* (Supplementary Figure S2C), *T. guizhouense* (Supplementary Figure S2E), *T. harzianum* (Supplementary Figure S2F), *T. virens* (Supplementary Figure S2G) and *T. koningiopsis* (Supplementary Figure S2I) showed relatively milder inhibition.

In terms of mycelial morphology, *Trichoderma* mycelia demonstrated the ability to overgrow and spread on *G. sichuanense* mycelia, leading to the formation of irregular conidial clusters (Supplementary Figure S2). This resulted in the gradual withering of *G. sichuanense* mycelia. In some cases, certain *Trichoderma* strains completely covered the *G. sichuanense* mycelium with their spores. Additionally, we observed various pigments and antagonistic streaks on the back of the culture medium (Supplementary Figure S2).

### Fungicide sensitivity of isolates and *G. sichuanense*

In order to assess the effectiveness of fungicides against green mold disease, we conducted tests using six different fungicides in this study. Initially, we selected nine *Trichoderma* isolates to evaluate their sensitivity to these fungicides. The results showed that the inhibitory effect of the fungicides on *Trichoderma* growth varied, with stronger inhibition observed at higher fungicide concentrations. Table 4 presents the results, highlighting that prochloraz-manganese exhibited the highest inhibitory effect among the tested fungicides, as indicated by its minimum EC50 value, while Mancozeb showed the weakest inhibition with the highest EC50 value.

**Table 4:** Mean effective concentration to cause inhibition of by 50% (EC50) values of nine *Trichoderma* isolates from China to six fungicides.

Isolates	EC50 (μg mL^−1^)					
	Mancozeb	Chlorothalonil	Fludioxinil	Prochloraz	Carbendazim	Prochlorza-Mn
*T. ganodermatiderum*	78.81 ± 0.0245	3.381 ± 0.00137	2.786 ± 0.0012	0.0069 ± 0.0001	0.0086 ± 0.0001	0.0013 ± 0.0001
*T. citrinoviride*	180.4 ± 0.7359	8.9910 ± 0.0008	0.0445 ± 0.0012	0.0519 ± 0.0010	0.0301 ± 0.0008	0.0040 ± 0.0008
*T. hamatum*	47.51 ± 0.3764	0.0469 ± 0.0009	0.0029 ± 0.0006	0.0033 ± 0.0005	0.0059 ± 0.0005	0.0014 ± 0.0006
*T. asperellum*	129.1 ± 0.3764	0.5085 ± 0.0006	0.0377 ± 0.0004	0.0342 ± 0.0008	0.0073 ± 0.0006	0.0051 ± 0.0005
*T. guizhouense*	4.375 ± 0.1256	4.907 ± 0.0006	103.4 ± 0.9908	0.7338 ± 0.0079	0.0107 ± 0.0071	0.0047 ± 0.0015
*T. virens*	29.04 ± 0.1059	0.3593 ± 0.0135	139.6 ± 0.1351	0.0125 ± 0.0078	0.0073 ± 0.0107	0.0031 ± 0.0061
*T. paratroviride*	101.9 ± 0.0522	0.0462 ± 0.0062	0.0286 ± 0.0069	0.8578 ± 0.0005	0.0131 ± 0.0023	0.0082 ± 0.0009
*T. harzianum*	114.2 ± 0.0482	0.0082 ± 0.0002	105.9 ± 0.0157	0.0061 ± 0.0002	0.0155 ± 0.0002	0.0045 ± 0.0001
*T. koningiopsis*	30.20 ± 0.0026	0.0067 ± 0.0001	0.0019 ± 0.0001	7.432 ± 0.0001	0.0683 ± 0.0002	0.0051 ± 0.0001
*G. sichuanense*	11.06 ± 0.0019	3.622 ± 0.0002	0.1211 ± 0.0002	3.443 ± 0.0003	8.573 ± 0.0002	17.22 ± 0.0002

Furthermore, we evaluated the inhibitory effects of the fungicides on the growth of *G. sichuanense* mycelium through extensive tests. Significant variations in the effects of the six fungicides on the growth of *G. sichuanense* mycelium were observed, which generally aligned with the effects observed on *Trichoderma* strains. Notably, prochloraz-manganese had the least impact on the growth of *G. sichuanense* mycelium, displaying the highest EC50 value while exhibiting the strongest inhibitory effect on *Trichoderma* mycelium (Table 4). These findings suggest that low concentrations of prochloraz-manganese can be effective in controlling *Trichoderma*. Additionally, prochloraz and carbendazim demonstrated good inhibitory effects on all *Trichoderma* strains (Supplementary Figures S3–S11).

## Discussion

Green mold disease caused by *Trichoderma* species poses significant challenges in *G. sichuanense* cultivation, leading to economic losses and hindering industry growth (Lu et al., 2016; Huang et al., 2018; Zhang et al., 2018; Cai et al., 2020; An et al., 2022). This study aimed to comprehensively investigate *Trichoderma* species associated with *G. sichuanense*, focusing on their identification, characterization, pathogenicity assessment, and evaluation of fungicide efficacy. By addressing these objectives, we aimed to provide valuable insights into disease management strategies and the development of effective control measures for *G. sichuanense* cultivation.

Through morphological and molecular analyses, we successfully identified nine *Trichoderma* species associated with *G. sichuanense*: *T. asperellum, T. citrinoviride, T. ganodermatiderum, T. guizhouense, T. hamatum, T. harzianum, T. koningiopsis, T. paratroviride, and T. virens* (Lu et al., 2016; Huang et al., 2018; Zhang et al., 2018; Cai et al., 2020; An et al., 2022). These findings contribute to our understanding of the diversity and population dynamics of *Trichoderma* species associated with *G. sichuanense*, providing valuable insights for further research and disease management strategies.

A comprehensive understanding of the diversity and distribution of *Trichoderma* species in *G. sichuanense* cultivation is crucial for developing effective strategies to manage green mold disease. Our study revealed a wide range of *Trichoderma* species associated with green mold disease in *G. sichuanense*, including species known to affect mushrooms worldwide. Previous studies have also identified *Trichoderma* species as causative agents of green mold disease in various mushroom hosts, such as *Agaricus bisporus*, *Pleurotus ostreatus*, *Lentinula edodes*, *Flammulina filiformis*, *Tricholoma matsutake*, and *Dictyophora rubrovolvata* (Choi et al., 1998; Kosanović et al., 2015, 2020; Wang et al., 2016; Seung et al., 2018; Innocenti et al., 2019; Chen et al., 2021). These findings emphasize the broad host range of *Trichoderma* species and their significant economic impact on global mushroom cultivation.

Comparing our results with previous studies on *Trichoderma* species associated with *G. sichuanense*, we confirmed the presence of several previously reported species, including *T. asperellum*, *T. citrinoviride*, *T. guizhouense*, *T. hamatum*, *T. paratroviride*, and *T. virens* (Bissett et al., 2015; Zhu et al., 2017; An et al., 2022). However, our identification of *T*. *ganodermatiderum* in this specific cultivation system confirms its previously reported association as a pathogen on *G*. *sichuanense* (An et al., 2022). The incorporation of molecular data, specifically TEF1-a and RPB2 gene sequences, in our identification process significantly enhanced the accuracy and reliability of species identification. This approach contributes to a more comprehensive understanding of *Trichoderma* populations in *G. sichuanense* cultivation and adds to the growing body of knowledge on *Trichoderma* diversity associated with specific host plants.

The confrontation assay demonstrated that *Trichoderma* species effectively overgrow and spread on *G. sichuanense* mycelia, leading to the formation of irregular conidial clusters and the gradual withering of the mycelia. Some *Trichoderma* strains completely covered the *G. sichuanense* mycelium with their spores. The presence of pigments and antagonistic streaks on the culture medium further confirmed the antagonistic behavior of *Trichoderma* species against *G. sichuanense*. These findings indicate that the identified *Trichoderma* species have the ability to suppress the growth of *G. sichuanense* mycelia and can be considered as pathogens causing green mold disease in *G. sichuanense*. The pigments produced by *Trichoderma* species may play a role in their antagonistic behavior against *G. sichuanense* by acting as defense mechanisms, allowing *Trichoderma* to outcompete and suppress the growth of *G. sichuanense* mycelia (Kubicek et al., 2019; Robinson, 2022).

Further research should focus on investigating the specific mechanisms underlying the inhibition of *G. sichuanense* mycelial growth by *Trichoderma* species, as well as characterizing and understanding the role of the pigments and metabolites produced by *Trichoderma*. Such studies will provide valuable insights into the interaction between *Trichoderma* and *G. sichuanense* and contribute to the development of effective strategies for managing green mold disease.

The effectiveness of fungicides in controlling green mold disease caused by *Trichoderma* species is crucial for successful mushroom cultivation. In this study, we evaluated the efficacy of six different fungicides against *Trichoderma* growth and their inhibitory effects on *G. sichuanense* mycelium. Our results revealed varying levels of inhibition on *Trichoderma* growth by the tested fungicides. The inhibitory effect was stronger at higher fungicide concentrations (Table 4). Prochloraz-manganese exhibited the highest inhibitory effect, as evidenced by its minimum EC50 value (Shamshad et al., 2009), while Mancozeb showed the weakest inhibition with the highest EC50 value. These findings highlight the dependence of fungicide effectiveness on both the specific fungicide used and its concentration. Furthermore, we investigated the effects of the fungicides on the growth of *G. sichuanense* mycelium. Interestingly, the inhibitory effects of the six fungicides on *G. sichuanense* mycelium generally aligned with their effects on *Trichoderma* strains. Notably, despite exhibiting the strongest inhibitory effect on *Trichoderma* mycelium, prochloraz-manganese had the least impact on *G. sichuanense* mycelium growth (Table 4; Hatvani, 2008; Grogan and Jukes, 2010). This suggests that prochloraz-manganese can effectively control *Trichoderma* without severely affecting the growth of *G. sichuanense* mycelium, even at low concentrations.

The results indicate that prochloraz and carbendazim exhibited strong inhibitory effects on all tested Trichoderma strains (Supplementary Figure S3–S11), suggesting their potential as broad-spectrum fungicides for controlling *Trichoderma* species in mushroom cultivation (Innocenti et al., 2019). These findings underscore the importance of selecting fungicides based on their specific inhibitory effects on Trichoderma species, taking into account their compatibility with the growth of the mushroom host. Notably, prochloraz-manganese, prochloraz, and carbendazim have shown promise in effectively managing Trichoderma growth while minimizing their impact on *G*. *sichuanense* mycelium.

However, it is important to consider the potential development of fungicide resistance and the long-term sustainability of fungicide use in disease management strategies. To mitigate the economic losses associated with green mold disease while minimizing negative impacts on mushroom production and the environment (Potocnik et al., 2015), alternative control measures and integrated disease management approaches should be explored. These measures can incorporate cultural practices and biological control agents. Such approaches would enhance the sustainability of mushroom cultivation and reduce reliance on fungicides.

There are several limitations to consider in this study. Firstly, the survey was conducted in a specific geographic region of China, including Zhejiang, Hubei, and Jilin Province. Therefore, the findings may not be representative of the entire country or other regions where *G*. *sichuanense* is cultivated. Further studies in different regions and countries would provide a more comprehensive understanding of the prevalence and diversity of *Trichoderma* species causing green mold disease in *G*. *sichuanense*. Secondly, while morphological and phylogenetic analysis were employed to classify the isolated *Trichoderma* strains into different species, these methods have certain limitations. Additional molecular techniques, such as DNA sequencing or genotyping, would provide more precise identification and a deeper understanding of the genetic diversity and relationships among the *Trichoderma* pathogens.

Furthermore, this study focused primarily on the pathogenicity of the identified *Trichoderma* species through inoculation tests on healthy *G*. *sichuanense* fruiting bodies. The investigation of other factors influencing the disease development, such as environmental conditions, host resistance, or interactions with other microorganisms, was not extensively explored. A more comprehensive study incorporating these factors would provide a more holistic understanding of green mold disease in *G*. *sichuanense*. Lastly, the sensitivity of the *Trichoderma* species to fungicides was assessed using a limited number of commercially available fungicides. The evaluation of additional fungicides or alternative management approaches would contribute to a more comprehensive understanding of effective control measures for green mold disease. Addressing these limitations in future research endeavors would help to enhance our understanding of the prevalence, genetic diversity, pathogenicity mechanisms, and effective management strategies for *Trichoderma* species causing green mold disease in *G*. *sichuanense*.

In summary, our study provides valuable insights into the host range of *Trichoderma* species associated with *G. sichuanense* and their susceptibility to *T. guizhouense*, *T. virens*, *T. hamatum*, *T. paratroviride*, *T. asperellum*, and *T. citrinoviride*. Furthermore, we have evaluated the effectiveness of selected fungicides in controlling green mold disease, offering valuable information for disease prevention and management in edible fungi. These findings are of significant importance for the effective control of green mold disease on *G. sichuanense* in China. our study also contributes to the existing knowledge on the effectiveness of fungicides against *Trichoderma* and their impact on *G. sichuanense* mycelium. These findings provide a foundation for the development of robust disease management strategies and underscore the importance of continued research to enhance the sustainability of mushroom cultivation. By understanding the sensitivity of *Trichoderma* strains and the efficacy of fungicides, we can develop targeted strategies for disease management. However, it is crucial to conduct further research to explore sustainable approaches that minimize potential fungicide resistance and environmental impacts in mushroom cultivation.

## Data availability statement

The data presented in the study are deposited in the NCBI repository. TEF-1a sequence accession numbers: OR291383-OR291398; RPB2 sequence accession numbers: OR291399-OR291417; ITS sequence accession numbers: OR569141-OR569159.

## Author contributions

XuL: Conceptualization, Data curation, Formal analysis, Investigation, Methodology, Resources, Software, Validation, Visualization, Writing – original draft, Writing – review & editing. FS: Formal analysis, Methodology, Resources, Software, Visualization, Writing – review & editing. YT: Data curation, Investigation, Methodology, Resources. JH: Conceptualization, Methodology, Resources, Software. QW: Methodology, Validation. SL: Methodology, Validation. NR: Methodology, Validation. MW-K: Formal analysis, Visualization. CL: Investigation, Resources, Visualization. BZ: Funding acquisition, Project administration, Supervision, Visualization, Writing – review & editing. XiL: Conceptualization, Investigation, Project administration, Resources, Supervision, Visualization, Writing – review & editing. YL: Conceptualization, Funding acquisition, Project administration, Supervision, Visualization, Writing – review & editing.

## Footnotes

^1^ https://www.ncbi.nlm.nih.gov/BLAST/

^2^ http://www.ncbi.nlm.nih.gov/genbank

^3^ http://tree.bio.ed.ac.uk/software/figtree/

## References

[ref1] An X. Y. Cheng G. H. Gao H. X. Li X. F. Yang Y. Li D.. (2022). Phylogenetic analysis of *Trichoderma* species associated with green mold disease on mushrooms and two new pathogens on *Ganoderma sichuanense*. J. Fungi 8:704. doi: 10.3390/jof8070704, PMID: 35887460 PMC9318549

[ref2] Bissett J. (1984). A revision of the genus *Trichoderma*. I. Section *Longibrachiatum* sect. nov. Can. J. Bot. 62, 924–931. doi: 10.1139/b84-131

[ref3] Bissett J. Gams W. Jaklitsch W. Samuels G. J. (2015). Accepted *Trichoderma* names in the year 2015. IMA Fungus 6, 263–295. doi: 10.5598/imafungus.2015.06.02.02, PMID: 26734542 PMC4681254

[ref4] Cai F. Druzhinina I. S. (2021). In honor of John Bissett: authoritative guidelines on molecular identification of *Trichoderma*. Fungal Divers. 107, 1–69. doi: 10.1007/s13225-020-00464-4

[ref5] Cai M. Idrees M. Zhou Y. Zhang C. Xu J. (2020). First report of green mold disease caused by *Trichoderma hengshanicum* on *Ganoderma lingzhi*. Mycobiology 48:427-430. doi: 10.1080/12298093.2020.1794230 33177923 PMC7580564

[ref6] Chaverri P. Branco-Rocha F. Jaklitsch W. Gazis R. Degenkolb T.. (2015). Systematics of the *Trichoderma harzianum* species complex and the re-identification of commercial biocontrol strains. Mycologia 107, 558–590. doi: 10.3852/14-147 25661720 PMC4885665

[ref7] Chaverri P. Castlebury L. A. Samuels G. J. Geiser D. M. (2003). Multilocus phylogenetic structure within the *Trichoderma harzianum*/*Hypocrea lixii* complex. Mol. Phylogenet. Evol. 27, 302–313. doi: 10.1016/S1055-7903(02)00400-1, PMID: 12695093

[ref8] Chen X. Y. Zhou X. H. Zhao J. Tang X. L. Pasquali M. Migheli Q.. (2021). Occurrence of green mold disease on *Dictyophora rubrovolvata* caused by *Trichoderma koningiopsis*. J. Plant Pathol. 103, 981–984. doi: 10.1007/s42161-021-00861-x

[ref9] Chen K. Zhuang W. Y. (2017). Discovery from a large-scaled survey of *Trichoderma* in soil of China. Sci. Rep. 7:9090. doi: 10.1038/s41598-017-07807-3 28831112 PMC5567330

[ref1001] China Edible Fungi Association (2020). Available at: http://www.cefa.org.cn/web/index.html

[ref10] Chiu H. F. Fu H. Y. Lu Y. Y. Han Y. C. Shen Y. C. Venkatakrishnan K.. (2017). Triterpenoids and polysaccharide peptides-enriched *Ganoderma lucidum*: a randomized, double-blind placebo-controlled crossover study of its antioxidation and hepatoprotective efficacy in healthy volunteers. Pharm. Biol. 55, 1041–1046. doi: 10.1080/13880209.2017.1288750, PMID: 28183232 PMC6130508

[ref11] Choi I. Y. Lee W. H. Choi J. S. (1998). Forest green mold disease caused by *Trichoderma pseudokoningii* in winter mushroom, *Flammulina velutipes*. Korean J. Mycol. 26, 531–537.

[ref12] Edgar R. C. (2004). MUSCLE: multiple sequence alignment with high accuracy and high throughput. Nucleic Acids Res. 32, 1792–1797. doi: 10.1093/nar/gkh340, PMID: 15034147 PMC390337

[ref13] Etebarian H. R. Sholberg P. L. Eastwell K. C. Sayler R. J. (2005). Biological control of apple blue mold with *Pseudomonas fluorescens*. Can. J. Microbiol. 51, 591–598. doi: 10.1139/w05-039 16175208

[ref14] Gams W. Bissett J. (1998). Morphology and identification of *Trichoderma*. Trichoderma Gliocladium 1, 3–34.

[ref15] Grogan H. M. Jukes A. A. (2010). Persistence of the fungicides thiabendazole, carbendazim and prochloraz-Mn in mushroom casing soil. Pest Manag. Sci. 59, 1225–1231. doi: 10.1002/ps.759 14620049

[ref16] Guindon S. Dufayard J. F. Lefort V. Anisimova M. Hordijk W. Gascuel O. (2010). New algorithms and methods to estimate maximum-likelihood phylogenies: assessing the performance of PhyML 3.0. Syst. Biol. 59, 307–321. doi: 10.1093/sysbio/syq010, PMID: 20525638

[ref17] Hall T. A. (1999). Bioedit: a user-friendly biological sequence alignment editor and analysis program for windows 95/98/Nt. Nucleic Acids Symp. 41, 95–98. doi: 10.1021/bk-1999-0734.ch008

[ref18] Hall T. A. (2011). BioEdit: an important software for molecular biology. Gerf Bull Biosci. 2, 60–61.

[ref19] Hatvani L. (2008). Mushroom pathogenic *Trichoderma* species: occurrence, diagnosis and extracellular enzyme production. PhD thesis. Szeged, Hungary: University of Szeged

[ref20] Huang X. Madan A. (1999). CAP3: a DNA sequence assembly program. Genome Res. 9, 868–877. doi: 10.1101/gr.9.9.868, PMID: 10508846 PMC310812

[ref21] Huang X. W. Yan Y. H. Zhang T. Geng L. J. Cheng Z.. (2018). Isolation, identification and rapid detection of the pathogen causing green mold on *Ganoderma lingzhi*. J. Plant Prot. 45, 1435–1436. (in Chinese)

[ref22] Innocenti G. Montanari M. Righini H. Roberti R. (2019). *Trichoderma* species associated with green mould disease of *Pleurotus ostreatus* and their sensitivity to prochloraz. Plant Pathol. 68. doi: 10.1111/ppa.12953

[ref23] Jaklitsch M. W. (2009). European species of Hypocrea. Part I. The green-spored species. Stud. Mycol. 63, 1–91. doi: 10.3114/sim.2009.63.01 19826500 PMC2757427

[ref24] Jaklitsch W. M. Komon M. Kubicek C. P. Druzhinina I. S. (2005). *Hypocrea voglmayrii* sp. nov. from the Austrian Alps represents a new phylogenetic clade in *Hypocrea/Trichoderma*. Mycologia 97, 1365–1378. doi: 10.3852/mycologia.97.6.1365 16722227

[ref25] Kang H. J. Sigler L. Lee J. Gibas C. F. Yun S. H.. (2010). *Xylogone ganodermophthora* sp. nov., an ascomycetous pathogen causing yellow rot on cultivated mushroom *Ganoderma lucidum* in Korea. Mycologia 102, 1167–1184. doi: 10.3852/09-304 20943517

[ref26] Kim C. H. Hassan O. Chang T. (2020). Diversity, pathogenicity, and fungicide sensitivity of *Colletotrichum* species associated with apple anthracnose in South Korea. Plant Dis. 104, 2866–2874. doi: 10.1094/PDIS-01-20-0050-RE, PMID: 32924872

[ref27] Kosanović D. Grogan H. Kavanagh K. (2020). Exposure of *Agaricus bisporus* to *Trichoderma aggressivum* f. *europaeum* leads to growth inhibition and induction of an oxidative stress response. Fungal Biol. 124, 814–820. doi: 10.1016/j.funbio.2020.07.003, PMID: 32883431

[ref28] Kosanović D. Potočnik I. Vukojević J. Stajić M. Rekanović E. Stepanović M.. (2015). Fungicide sensitivity of *Trichoderma* spp. from *Agaricus bisporus* farms in Serbia. J. Environ. Sci. Health B 50, 607–613. doi: 10.1080/03601234.2015.1028849, PMID: 26065521

[ref29] Krobthong S. Choowongkomon K. Suphakun P. Kuaprasert B. Yingchutrakul Y. (2021). The anti-oxidative effect of Lingzhi protein hydrolysates on lipopolysaccharide-stimulated A549 cells. Food Biosci. 41:101093. doi: 10.1016/j.fbio.2021.101093

[ref30] Kubicek C. P. Steindorff A. S. Chenthamara K. Manganiello G. Henrissat B.. (2019). Evolution and comparative genomics of the most common *Trichoderma* species. BMC Genomics 20:485. doi: 10.1186/s12864-019-5680-7 31189469 PMC6560777

[ref31] Lanfear R. Frandsen P. B. Wright A. M. Senfeld T. Calcott B. (2017). PartitionFinder 2: new methods for selecting partitioned models of evolution for molecular and morphological phylogenetic analyses. Mol. Biol. Evol. 34, 772–773. doi: 10.1093/molbev/msw260, PMID: 28013191

[ref32] Liu Y. J. Whelen S. Hall B. D. (1999). Phylogenetic relationships among ascomycetes: evidence from an RNA polymerse II subunit. Mol. Biol. Evol. 1799-1808. doi: 10.1093/oxfordjournals.molbev.a026092 10605121

[ref33] Lu B. H. Zuo B. Liu X. L. Feng J. Wang Z. M. Gao J. (2016). *Trichoderma harzianum* causing green mold disease on cultivated *Ganoderma lucidum* in Jilin province, China. Plant Disease 100:2524. doi: 10.1094/PDIS-04-16-0422-PDN

[ref35] Pan Y. Lin Z. B. (2019). Anti-aging effect of *Ganoderma* (Lingzhi) with health and fitness. Adv. Exp. Med. Biol. 1182, 299–309. doi: 10.1007/978-981-32-9421-9_13 31777025

[ref36] Park M. S. Bae K. S. Yu S. H. (2006). Two new species of *Trichoderma* associated with green mold of oyster mushroom cultivation in Korea. Mycobiology 34, 111–113. doi: 10.4489/MYCO.2006.34.3.111, PMID: 24039481 PMC3769556

[ref37] Potocnik I. Stepanovic M. Rekanovic E. Todorovic B. Milijasevic M. S. (2015). Disease control by chemical and biological fungicides in cultivated mushrooms: button mushroom, oyster mushroom and shiitake. Pestic. Phytomed 30, 201–208. doi: 10.2298/PIF1504201P

[ref38] Qiu Z. Zhong D. Yang B. (2019). Preventive and therapeutic effect of *Ganoderma* (Lingzhi) on liver injury. Adv. Exp. Med. Biol. 1182, 217–242. doi: 10.1007/978-981-32-9421-9_9 31777021

[ref39] Rahman M. A. Abdullah N. Aminudin N. (2018). Evaluation of the anti-oxidative and hypocholesterolemic effects of Lingzhi or Reishi medicinal mushroom, *Ganoderma lucidum* (Agaricomycetes), in ameliorating cardiovascular diseases. Int. J. Med. Mushrooms 20, 961–969. doi: 10.1615/IntJMedMushrooms.2018028370, PMID: 30806268

[ref40] Rahman M. A. Hossain S. Abdullah N. Aminudin N. (2020). Lingzhi or Reishi medicinal mushroom, *Ganoderma lucidum* (Agaricomycetes) ameliorates spatial learning and memory deficits in rats with hypercholesterolemia and alzheimer's disease. Int J Med Mushrooms. 22, 93–103. doi: 10.1615/IntJMedMushrooms.2020033383, PMID: 32464001

[ref41] Robinson S. C. (2022). Colorants produced by *Penicillium murcianum* are a natural moldicide against *Trichoderma* and other *Penicillium* species. Coatings 12:821. doi: 10.3390/coatings12060821

[ref42] Seaby A. (1987). Infection of mushroom compost by *Trichoderma* species. Mushroom J. 179, 355–361.

[ref43] Seung Y. O. Myung S. P. Hae J. C. Young W. L. (2018). Diversity and effect of *Trichoderma* isolated from the roots of *Pinus densiflora* within the fairy ring of pine mushroom (*Tricholoma matsutake*). PLoS One 13:e0205900. doi: 10.1371/journal.pone.0205900, PMID: 30403694 PMC6221287

[ref44] Shah S. Nasreen S. Kousar S. (2013). Efficacy of fungicides against *Trichoderma* spp. causing green mold disease of oyster mushroom (*Pleurotus sajor-caju*). Res. J. Microbiol. 8, 13–24. doi: 10.3923/jm.2013.13.24

[ref45] Shamshad A. Clift A. D. Mansfield S. (2009). Imazalil, manganese prochloraz and carbendazim treatments do not affect the yield of *Agaricus bisporus*, hybrid strain Sylvan A15 in New South Wales. Plant Prot. Quart. 24, 50–54. doi: 10.1586/1744666X.2016.1133295

[ref47] Wang G. Z. Cao X. T. Ma X. L. Guo M. P. Liu C. H. Yan L.. (2016). Diversity and effect of *Trichoderma* spp. associated with green mold disease on *Lentinula edodes* in China. Microbiology 5, 709–718. doi: 10.1002/mbo3.364, PMID: 27147196 PMC4985603

[ref48] Wang Y. Fan X. Wu X. (2020). *Ganoderma lucidum* polysaccharide (GLP) enhances antitumor immune response by regulating differentiation and inhibition of MDSCs via a CARD9-NF-κB-IDO pathway. Biosci. Rep. 40:BSR20201170. doi: 10.1042/BSR20201170 32530032 PMC7313449

[ref49] Wang X. C. Xi R. J. Li Y. Wang D. M. Yao Y. J. (2012). The species identity of the widely cultivated *Ganoderma*, ‘*G. lucidum*’ (Ling-zhi), in China. PLoS One 7:e40857. doi: 10.1371/journal.pone.0040857 22911713 PMC3401198

[ref50] Wong F. P. Midland S. L. (2007). Sensitivity distributions of California populations of *Colletotrichum cereale* to the DMI fungicides propiconazole, myclobutanil, tebuconazole, and triadimefon. Plant Dis. 91, 1547–1555. doi: 10.1094/PDIS-91-12-1547, PMID: 30780605

[ref51] Wu Q. Li Y. Peng K. Wang X. L. Ding Z. Y.. (2019). Isolation and characterization of three antihypertension peptides from the mycelia of *Ganoderma lucidum* (Agaricomycetes). J. Agric. Food Chem. 67, 1–11. doi: 10.1021/acs.jafc.9b02276 31246442

[ref52] Xiao C. Wu Q. Zhang J. Xie Y. Cai W.. (2016). Antidiabetic activity of *Ganoderma lucidum* polysaccharides F31 down-regulated hepatic glucose regulatory enzymes in diabetic mice. J. Ethnopharmacol. 196, 47–57. doi: 10.1016/j.jep.2016.11.044 27902927

[ref53] Yan Y. Xu J. Zhang C. Moodley O. Zhang L. (2019). Green mold on *Ganoderma lingzhi* (Agaricomycetes) caused by *Trichoderma atroviride*. Int. J. Med. Mushrooms 21, 515–521. doi: 10.1615/IntJMedMushrooms.2019030352

[ref54] Zhang D. Gao F. Jakovlić I. Zou H. Zhang J. Li W. X.. (2020). Phylosuite: an integrated and scalable desktop platform for streamlined molecular sequence data management and evolutionary phylogenetics studies. Mol. Ecol. Resour. 20, 348–355. doi: 10.1111/1755-0998.13096, PMID: 31599058

[ref55] Zhang T. Lu M. Z. Zhang C. L. Xu J. Z. (2018). First report of *Trichoderma longibrachiatum* causing green mold disease on *Ganoderma lingzhi*. Plant Dis. 103. doi: 10.1094/PDIS-05-18-0818-PDN

[ref56] Zhao J. D. Xu L. W. Zhang X. Q. (1983). Taxonomic studies on the family Ganodermataceae of China II. Acta Mycol. Sin. 2, 159–167. doi: 10.13346/j.mycosystema.1983.03.003 (In Chinese).

[ref57] Zhu Z. X. Xu H. X. Zhuang W. Y. Li Y. (2017). Two new green-spored species of *Trichoderma* (Sordariomycetes, Ascomycota) and their phylogenetic positions. MycoKeys 26, 61–75. doi: 10.3897/mycokeys.26.14919

[ref58] Zhu L. F. Yao Y. F. Ahmad Z. Chang M. W. (2019). Development of *Ganoderma lucidum* spore powder based proteoglycan and its application in hyperglycemic, antitumor and antioxidant function. Process Biochem. 2019, 103–111. doi: 10.1016/j.procbio.2019.05.025

[ref59] Zuo B. Lu B. H. Liu X. L. Wang Y. Ma G. L. Gao J. (2016). First report of *Cladobotryum mycophilum* causing cobweb on *Ganoderma lucidum* cultivated in Jilin Province, China. Plant Dis. 100:1239. doi: 10.1094/PDIS-12-15-1431-PDN

