# Supplementary figures and images for: Characterization and fungicide sensitivity of Trichoderma species causing green mold of Ganoderma sichuanense in China
Source: Front Microbiol. 2023 Oct 19;14:1264699. doi: 10.3389/fmicb.2023.1264699 (PMC10620716; doi:10.3389/fmicb.2023.1264699)

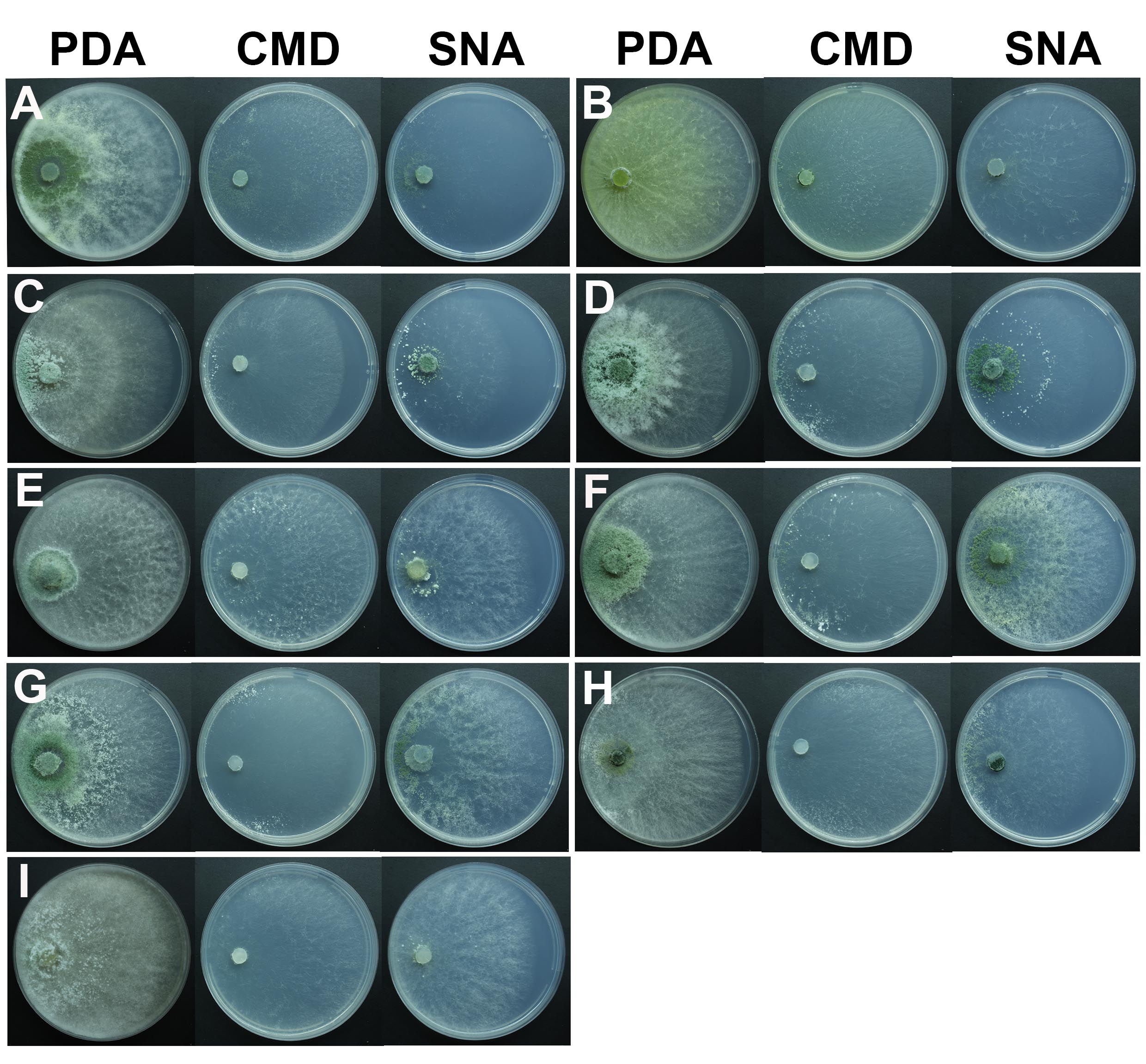

Supplement: Supplementary file 1 [file Figure_1.JPEG]

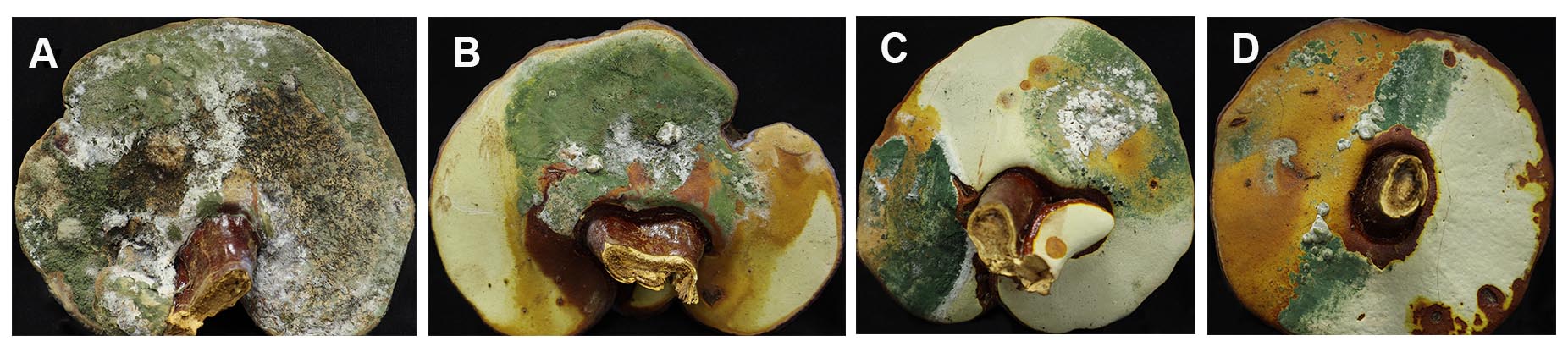

Supplement: Supplementary file 2 [file Figure_2.JPEG]

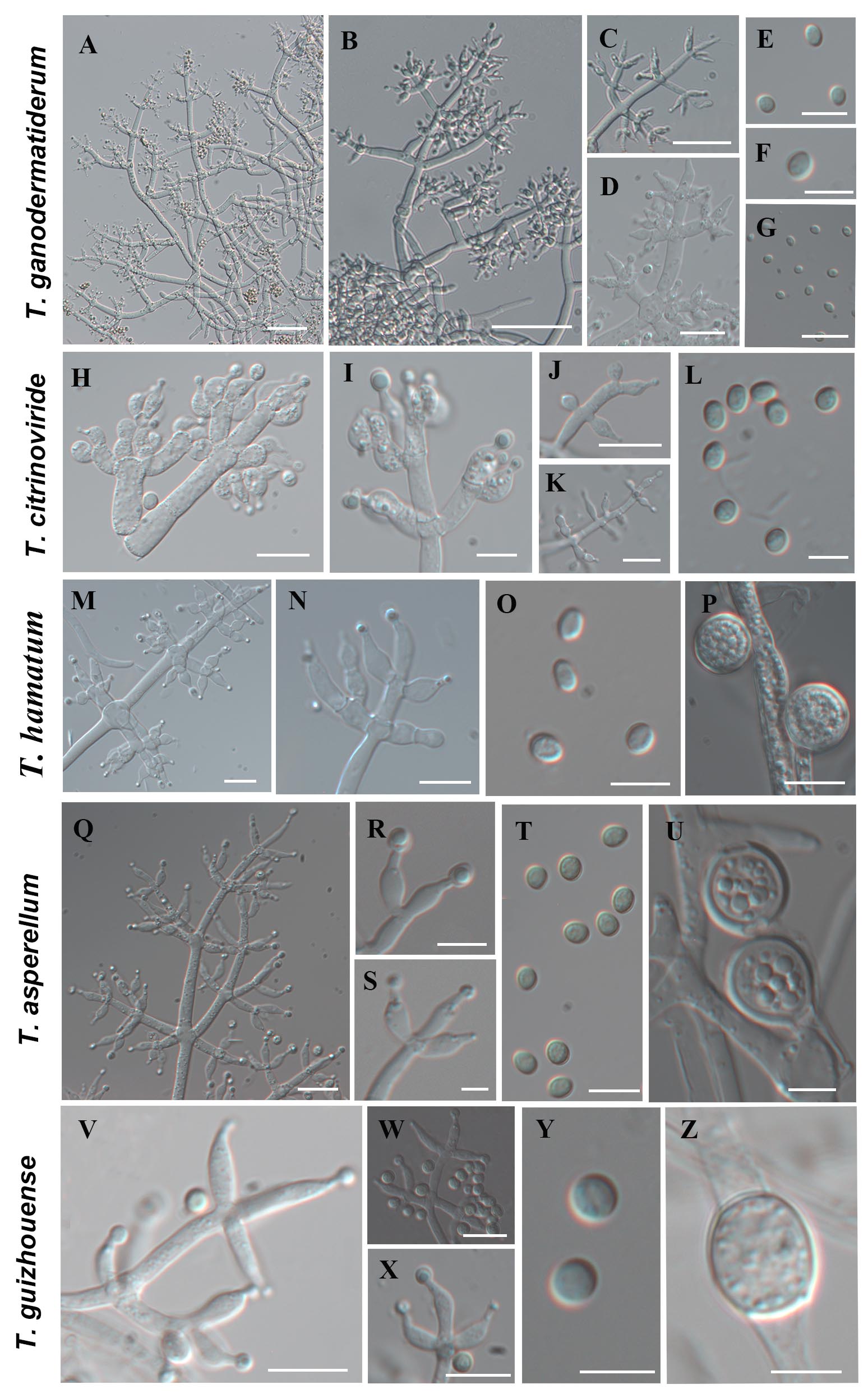

Supplement: Supplementary file 3 [file Figure_3.JPEG]

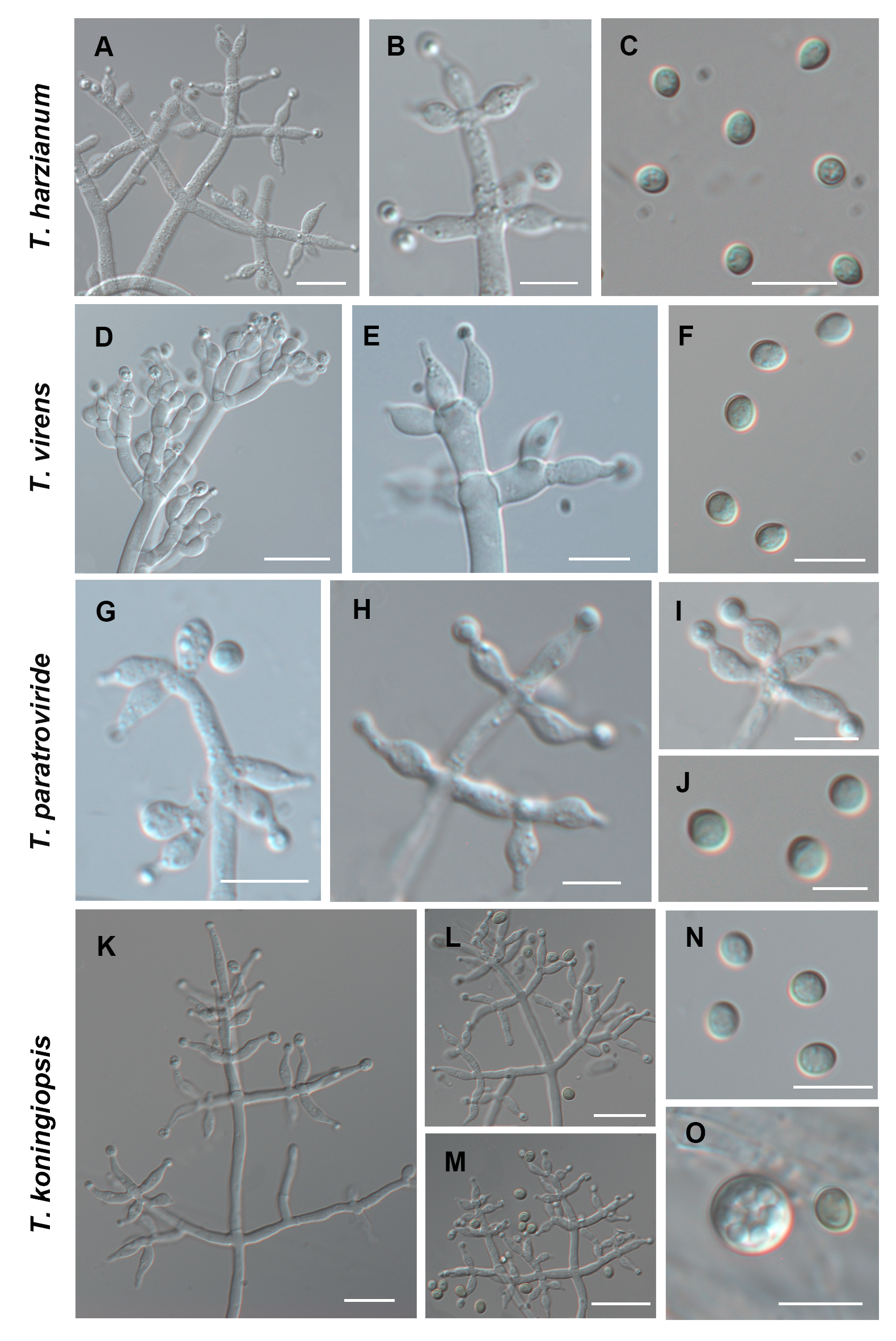

Supplement: Supplementary file 4 [file Figure_4.TIF]

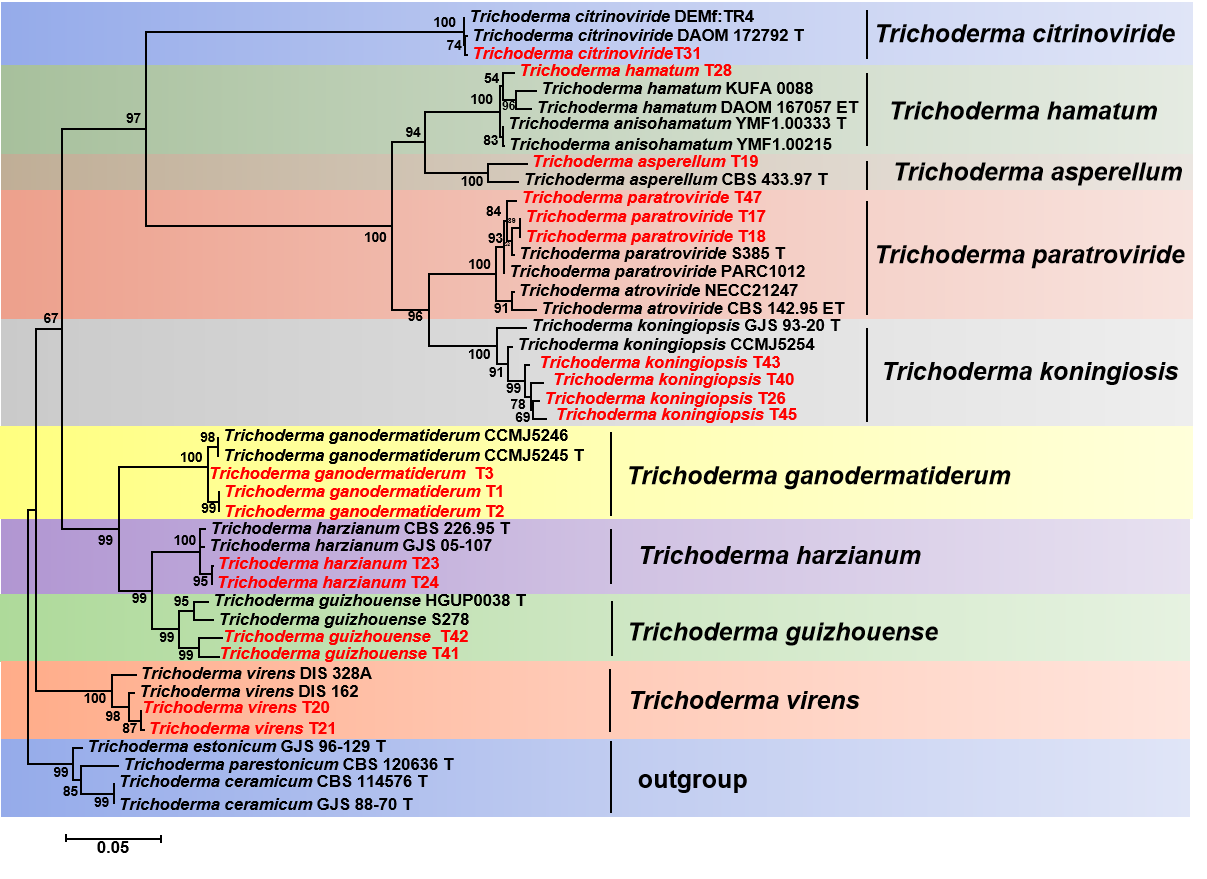

Supplement: Supplementary file 5 [file Figure_5.TIF]

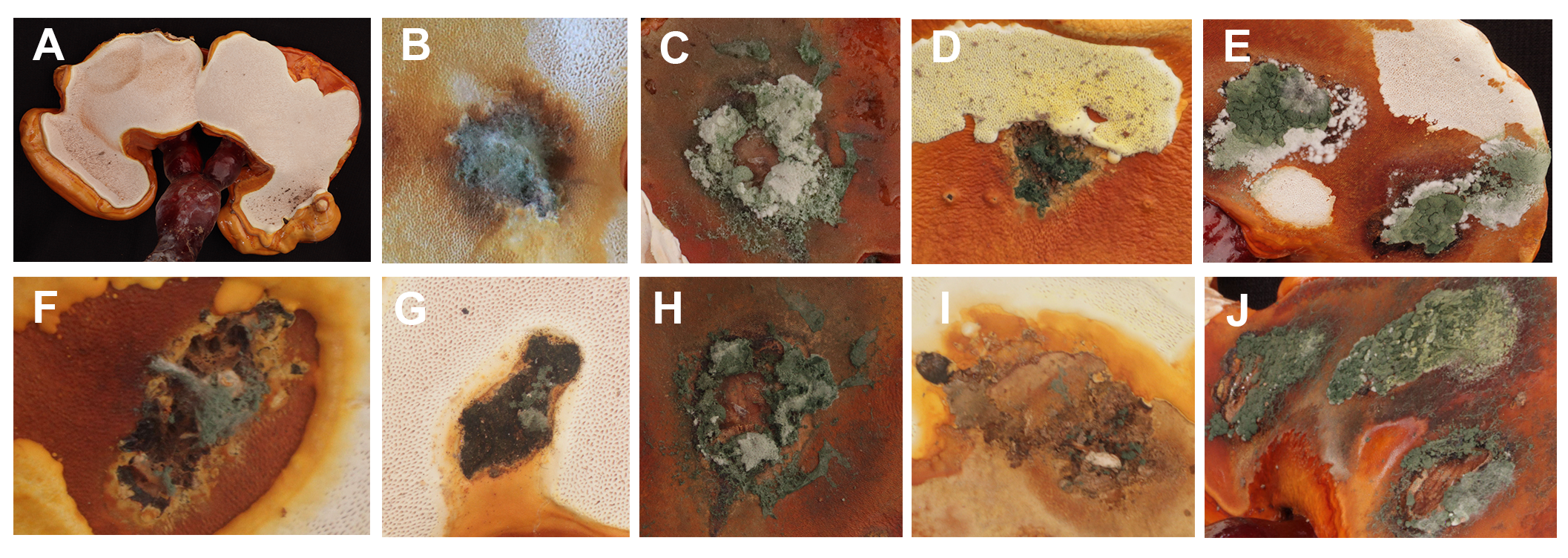

Supplement: Supplementary file 6 [file Figure_6.TIF]
