# Supplementary material for: Characterization and fungicide sensitivity of Trichoderma species causing green mold of Ganoderma sichuanense in China
Source: Front Microbiol. 2023 Oct 19;14:1264699. doi: 10.3389/fmicb.2023.1264699 (PMC10620716; doi:10.3389/fmicb.2023.1264699)
Supplement: Supplementary file 7 [file Data_Sheet_1.docx]

Table S1. Voucher/Specimen Numbers, country and their corresponding GenBank accession numbers of sequences used for phylogenetic analyses. Sequences produced in this study are in bold.

| Scientific Name | Voucher/Specimen Numbers | Country | Substrate | GenBank Accession Numbers | |
| --- | --- | --- | --- | --- | --- |
|  |  |  |  | RPB2 | TEF1-a |
| *T. afarasin* | GJS 99-227 T | Cameroon | Soil | / | AF348093 |
| *T. afarasin* | DIS 314F | Cameroon | *Cola* sp. | FJ442778 | FJ463400 |
| *T. afarasin* | GJS 06-98 | Cameroon | Soil | / | FJ463327 |
| *T. afarasin* | DIS 377A | Cameroon | *Cola* sp. | FJ442799 | FJ463322 |
| *T. afroharzianum* | LESF229 | Brazil | soil | KT278945 | KT279013 |
| *T. afroharzianum* | GJS 04-186 T | Peru | *Moniliophthora roreri* | FJ442691 | FJ463301 |
| *T. afroharzianum* | CBS 466.94 | Netherlands | / | KP009150 | KP008851 |
| *T. afroharzianum* | GJS 04-193 | Peru | *Moniliophthora roreri* | FJ442709 | FJ463298 |
| *T. aggregatum* | HMAS 248864 | China | Soil | KY688002 | KY688063 |
| *T. aggressivum* f*. europaeum* | CBS 100525 | / | Mushroom compost | AF545541 | AF348095 |
| *T. aggressivum* f. *aggressivum* | DAOM 222156 T | / | Mushroom compost | FJ442752 | AF348098 |
| *T. aggressivum* f. *europaeum* | CBS 100526 | Ireland | Mushroom compost | KP009166 | KP008993 |
| *T. alni* | CBS120633 T | England | *Alnus glutinosa* | EU498349 | EU498312 |
| *T. alni* | CPK2494 | / | / | EU498350 | EU498313 |
| *T. alni* | HMAS 252890 | China | / | KT343763 | KT343758 |
| *T. alpinum* | HMAS 248821 T | China | Soil | KY687958 | KY688012 |
| *T. alpinum* | HMAS 248830 | China | Soil | KY687961 | KY688015 |
| *T. alpinum* | HMAS 248870 | China | Soil | KY687963 | KY688017 |
| *T. amazonicum* | IB50 T | Peru | *Hevea brasiliensis* | HM142367 | HM142376 |
| *T. amazonicum* | IB95 | Peru | Hevea | HM142368 | HM142377 |
| *T. amazonicum* | LA265 | Peru | Hevea | HM142369 | HM142379 |
| *T. anisohamatum* | [YMF1.00333 T](https://www.ncbi.nlm.nih.gov/nuccore/MH177912.1) | China | / | MH155272 | MH177912 |
| *T. anisohamatum* | [YMF1.00253](https://www.ncbi.nlm.nih.gov/nuccore/MH262586.1) | China | / | MH262578 | MH236495 |
| *T. anisohamatum* | [YMF1.00215](https://www.ncbi.nlm.nih.gov/nuccore/MH262583.1) | China | / | MH262576 | MH236494 |
| *T. asperellum* | CBS 433.97 T | USA | soil | EU248617 | AY376058 |
| ***T. asperellum*** | **T19** | **China** | ***G. sichuanense*** | **OR291404** | **OR291385** |
| *T. atrobrunneum* | S3 | Italy | / | KJ665241 | KJ665376 |
| *T. atrobrunneum* | G.J.S. 92-110 T | [France](https://wi.knaw.nl/page/fungal_display/name/javascript:void(0)" \o "https://wi.knaw.nl/page/fungal_display/name/javascript:void(0)) | *Fagus sylvatica*, decorticated wood | / | AF443942 |
| *T. atrobrunneum* | GJS 05-101 | Italy | soil | FJ442745 | FJ463392 |
| *T. atrobrunneum* | G.J.S. 90-254 | / | / | / | AF443943 |
| *T. atrogelatinosum* | BMCC LU498 | New Zealand | / | / | KJ871087 |
| *T. atrogelatinosum* | CBS 237.63 ET | New Zealand | *Bondarzewia berkeleyi* | KJ842201 | / |
| *T. atrogelatinosum* | DAOM 167632 | / | / | / | KJ871083 |
| *[T. atroviride](https://www.ncbi.nlm.nih.gov/nuccore/MH862505.1)* | [CBS 142.95 ET](https://www.ncbi.nlm.nih.gov/nuccore/MH862505.1) | Slovenia | decayed log | EU341801 | AF456891 |
| *[T. atroviride](https://www.ncbi.nlm.nih.gov/nuccore/MH862505.1)* | NECC21247 | / | / | OL790433 | OL790432 |
| *[T. atroviride](https://www.ncbi.nlm.nih.gov/nuccore/MH862505.1)* | [T35](https://www.ncbi.nlm.nih.gov/nuccore/MW748227.1) | / | / | MW748227 | MW748225 |
| *T. azevedoi* | CEN1403 | Brazil | soil | MK696800 | MK696638 |
| *T. azevedoi* | CEN1422 | Brazil | soil | MK696821 | MK696660 |
| *T. bannaense* | HMAS 248840 T | China | Soil | KY687979 | KY688037 |
| *T. bannaense* | HMAS 248865 | China | Soil | KY688003 | KY688038 |
| *T. breve* | HMAS 248844 T | China | Soil | KY687983 | KY688045 |
| *T. breve* | HMAS 248845 | China | Soil | KY687984 | KY688046 |
| *T. brevicrassum* | HMAS 248871 T | China, | Soil | KY688008 | KY688064 |
| *T. brevicrassum* | HMAS 248872 | China | Soil | KY688009 | KY688065 |
| *T. brunneoviride* | CBS 120928 | / | / | EU498358 | EU498318 |
| *T. brunneoviride* | CBS 121130 T | / | / | EU498357 | EU498316 |
| *T. camerunense* | GJS 99-230 T | Cameroon | Soil | / | AF348107 |
| *T. camerunense* | GJS 99-231 | Cameroon | Soil | / | AF348108 |
| *T. catoptron* | DAOM 232830 ET | Sri Lanka | / | KJ842166 | KJ871245 |
| *T. catoptron* | GJS 02-76 ET | Sri Lanka | Wood | / | AY737726 |
| *T. ceramicum* | CBS 114576 T | [USA](https://wi.knaw.nl/page/fungal_display/name/javascript:void(0)" \o "https://wi.knaw.nl/page/fungal_display/name/javascript:void(0)) | wood | FJ860531 | FJ860628 |
| *T. ceramicum* | [GJS 88-70 T](https://www.ncbi.nlm.nih.gov/nuccore/AY737764.1) | [USA](https://wi.knaw.nl/page/fungal_display/name/javascript:void(0)" \o "https://wi.knaw.nl/page/fungal_display/name/javascript:void(0)) | wood | AF545510 | AF534593 |
| *T. cerinum* | BMCC LU784 | New Zealand | / | / | KJ871244 |
| *T. cerinum* | DAOM 230012 T | Nepal | / | KJ842184 | KJ871242 |
| *T. christiani* | S442 T | Spain | *Castanea sativa* | KJ665244 | KJ665439 |
| *T. christiani* | S93 | Italy | / | KJ665245 | KJ665442 |
| *T. cinnamomeum* | G.J.S. 96-128 | / | / | AY391916 | AY391977 |
| *T. cinnamomeum* | G.J.S. 97-233 | / | / | AY391919 | AY391978 |
| *T. cinnamomeum* | GJS 97-237 T | USA | Decaying wood | AY391920 | AY737732 |
| *T. citrinoviride* | [CBS 258.85 T](https://www.ncbi.nlm.nih.gov/nuccore/MH873564.1) | USA | forest soil under *Pinus* sp. | / | AY865637 |
| *T. citrinoviride* | [DAOM 172792 T](https://www.ncbi.nlm.nih.gov/nuccore/EU280036.1) | / | / | KJ842210 | KJ713208 |
| *T. citrinoviride* | [DEMf:TR4](https://www.ncbi.nlm.nih.gov/nuccore/OK422205.1) | Serbia | *Pinus sylvestris* bark | OK422202 | OK422205 |
| *T. citrinoviride* | [HZA9](https://www.ncbi.nlm.nih.gov/nuccore/MK962804.1) | China | soil | MK962804 | MK850831 |
| ***T. citrinoviride*** | **T31** | **China** | ***G. sichuanense*** | **OR291411** | **OR291392** |
| *T. compactum* | CBS 121218 T | China | soil | KF134789 | KF134798 |
| *T. concentricum* | HMAS 248833 T | Chinai | Soil | KY687971 | KY688027 |
| *T. concentricum* | HMAS 248858 | China | Soil | KY687997 | KY688028 |
| *T. corneum* | G.J.S. 97-82 | Thailand | bark | KJ665252 | KJ665455 |
| *T. endophyticum* | DIS 217A T | Ecuador | *Theobroma gileri* | / | FJ463319 |
| *T. endophyticum* | GS-2014a | Peru | *Hevea brasiliensis* | / | FJ967822 |
| *T. epimyces* | CBS 120534 T | Austria | *Fagus sylvatica* | EU498360 | EU498320 |
| *T. epimyces* | CPK1980 | / | / | EU498359 | EU498319 |
| *T. epimyces* | CPK2487 | / | / | EU498361 | EU498322 |
| *T. estonicum* | GJS 96-129 T | Estonia | *Hymenochaete tabacina* | AF545514 | AF534604 |
| *T. ganodermatiderum* | CCMJ5245 T | China | *G. Sichuanense* | ON567189 | ON567195 |
| *T. ganodermatiderum* | CCMJ5246 | China | *G. Sichuanense* | ON567190 | ON567196 |
| *T. ganodermatiderum* | CCMJ5247 | China | *G. Sichuanense* | ON567191 | ON567197 |
| *T. ganodermatiderum* | CCMJ5248 | China | *G. Sichuanense* | ON567192 | ON567198 |
| *T. ganodermatiderum* | CCMJ5249 | China | *G. Sichuanense* | ON567193 | ON567199 |
| *T. ganodermatiderum* | CCMJ5250 | China | *G. Sichuanense* | ON567194 | ON567200 |
| ***T. ganodermatiderum*** | **T1** | **China** | ***G. Sichuanense*** | **OR291399** | **OR291380** |
| ***T. ganodermatiderum*** | **T2** | **China** | ***G. Sichuanense*** | **OR291400** | **OR291381** |
| ***T. ganodermatiderum*** | **T3** | **China** | ***G. Sichuanense*** | **OR291401** | **OR291382** |
| *T. guizhouense* | HGUP0038 T | China | soil | JQ901400 | JN215484 |
| *T. guizhouense* | S628 | / | / | / | KJ665511 |
| *T. guizhouense* | S278 | Croatia | / | KF134791 | KF134799 |
| ***T. guizhouense*** | **T41** | **China** | ***G. Sichuanense*** | **OR291413** | **OR291394** |
| ***T. guizhouense*** | **T42** | **China** | ***G. Sichuanense*** | **OR291414** | **OR291395** |
| *T. hamatum* | [DAOM 167057 ET](https://www.ncbi.nlm.nih.gov/nuccore/EU279965.1) | Canada | / | AF545548 | EU279965 |
| *T. hamatum* | [KUFA 0088](https://www.ncbi.nlm.nih.gov/nuccore/OP250964.1) | / | / | OP250964 | OP250957 |
| *T. hamatum* | AJII | Brazil | soil | OM302359 | ON950423 |
| ***T. hamatum*** | **T28** | **China** | ***G. sichuanense*** | **OR291410** | **OR291391** |
| *T. harzianum* | CBS 226.95 T | England | Soil | AF545549 | AF348101 |
| *T. harzianum* | CBS 227.95 | / | / | / | AF348100 |
| *T. harzianum* | GJS 05-107 | Italy | *Ricinus communis* | FJ442708 | FJ463329 |
| *T. harzianum* | IMI 359823 | / | / | / | AF348092 |
| *T. harzianum* | GJS 04-71 | Italy | *Castanea sativa* | FJ442779 | FJ463396 |
| *T. harzianum* | Thaum12 | China | soil | MT118248 | MT081433 |
| ***T. harzianum*** | **T23** | **China** | ***G. Sichuanense*** | **OR291407** | **OR291388** |
| ***T. harzianum*** | **T24** | **China** | ***G. Sichuanense*** | **OR291408** | **OR291389** |
| *T. hausknechtii* | CBS 133493 T | France | / | KJ665276 | KJ665515 |
| *T. helicolixii* | S640 T | [Greece](https://wi.knaw.nl/page/fungal_display/name/javascript:void(0)" \o "https://wi.knaw.nl/page/fungal_display/name/javascript:void(0)) | *Spartium junceum* | KJ665278 | KJ665517 |
| *T. helicolixii* | S515 | Spain | / | KJ665277 | KJ665516 |
| *T. hengshanicum* | HMAS 248852 T | China | Soil | KY687991 | KY688054 |
| *T. hengshanicum* | HMAS 248853 | China | Soil | KY687992 | KY688055 |
| *T. hirsutum* | HMAS 248834 T | China | Soil | KY687972 | KY688029 |
| *T. hirsutum* | HMAS 248859 | / | / | KY687998 | KY688030 |
| *T. hunanense* | HMAS 248841 T | China | Soil | KY687980 | KY688039 |
| *T. hunanense* | HMAS 248867 | / | / | KY688005 | KY688040 |
| *T. ingratum* | HMAS 248822 T | China | Soil | KY687973 | KY688018 |
| *T. ingratum* | HMAS 248827 | / | / | KY687966 | KY688021 |
| *T. ingratum* | HMAS 248873 | / | / | KY688010 | KY688022 |
| *T. inhamatum* | CBS 273.78 T | Colombia | Soil | FJ442725 | AF348099 |
| *T. italicum* | CBS 132567 T | Italy | / | KJ665282 | KJ665525 |
| *T. italicum* | S15 | Italy | / | KJ665283 | KJ665526 |
| *T. koningii* | [CBS 457.96 ET](https://www.ncbi.nlm.nih.gov/nuccore/MH862585.1) | [Netherlands](https://wi.knaw.nl/page/fungal_display/name/javascript:void(0)" \o "https://wi.knaw.nl/page/fungal_display/name/javascript:void(0)) | soil | / | AF456909 |
| *T. koningii* | ATCC 64262 | Hungary | cork | / | AY376046 |
| *[T. koningiopsis](https://www.ncbi.nlm.nih.gov/nuccore/NR_131281.1)* | [GJS 93-20 T](https://www.ncbi.nlm.nih.gov/nuccore/DQ313140.1) | Cuba | branch | EU241506 | DQ284966 |
| *[T. koningiopsis](https://www.ncbi.nlm.nih.gov/nuccore/NR_131281.1)* | [Z2](https://www.ncbi.nlm.nih.gov/nuccore/MZ361069.1) | India | soil; apple rhizosphere | MZ361069 | MZ322893 |
| *[T. koningiopsis](https://www.ncbi.nlm.nih.gov/nuccore/NR_131281.1)* | [PR3](https://www.ncbi.nlm.nih.gov/nuccore/MZ322890.1) | India | apple rhizosphere | MZ361070 | MZ322890 |
| *[T. koningiopsis](https://www.ncbi.nlm.nih.gov/nuccore/NR_131281.1)* | [CCMJ5254](https://www.ncbi.nlm.nih.gov/nuccore/ON567202.1) | China | *G. Sichuanense* | ON567202 | ON567188 |
| *[T. koningiopsis](https://www.ncbi.nlm.nih.gov/nuccore/NR_131281.1)* | [CCMJ5253](https://www.ncbi.nlm.nih.gov/nuccore/ON567187.1) | China | *G. Sichuanense* | ON567201 | ON567187 |
| ***[T. koningiopsis](https://www.ncbi.nlm.nih.gov/nuccore/NR_131281.1)*** | **T26** | **China** | ***G. sichuanense*** | **OR291409** | **OR291390** |
| ***[T. koningiopsis](https://www.ncbi.nlm.nih.gov/nuccore/NR_131281.1)*** | **T40** | **China** | ***G. sichuanense*** | **OR291412** | **OR291393** |
| ***[T. koningiopsis](https://www.ncbi.nlm.nih.gov/nuccore/NR_131281.1)*** | **T43** | **China** | ***G. sichuanense*** | **OR291415** | **OR291396** |
| ***[T. koningiopsis](https://www.ncbi.nlm.nih.gov/nuccore/NR_131281.1)*** | **T45** | **China** | ***G. sichuanense*** | **OR291416** | **OR291397** |
| *T. lentiforme* | GJS 98-6 T | French Guiana | Decorticated wood | / | AF469195 |
| *T. lentiforme* | DIS 253B | Ecuador | *Theobroma cacao* | FJ442756 | FJ851875 |
| *T. lentiforme* | DIS 94D | Peru | *Theobroma* | FJ442749 | FJ463379 |
| *T. lentiforme* | DIS 167C | Brazil | *T. cacao* | FJ442689 | FJ463309 |
| *T. lentiforme* | DIS 218e | Ecuador | Ecuador | FJ442793 | FJ463310 |
| *T. lentinulae* | CGMCC 3.19847 T | China | *Lentinus edodes* | MN605867 | MN605878 |
| *T. lentinulae* | CGMCC 3.19848 | China | *L. edodes* | MN605868 | MN605879 |
| *T. lentinulae* | CGMCC 3.19849 | China | *L. edodes* | MN605869 | MN605880 |
| *T. lentinulae* | CGMCC 3.19699 | China | Soil | MN605876 | MN605887 |
| *T. lentinulae* | CGMCC 3.19700 | China | Soil | MN605877 | MN605888 |
| *T. liberatum* | HMAS 248831 T | China | Soil | KY687969 | KY688025 |
| *T. liberatum* | HMAS 248832 | China | Soil | KY687970 | KY688026 |
| *T. linzhiense* | HMAS 248846 T | China | Soil | KY687985 | KY688047 |
| *T. linzhiense* | HMAS 248874 | China | Soil | KY688011 | KY688048 |
| *T. lixii* | G.J.S. 97-96 ET | Thailand | Decayed *Ganoderma* | KJ665290 | AF443938 |
| *T. lixii* | G.J.S 05-82 | / | / | / | EF191326 |
| *T. neotropicale* | LA11 | / | / | / | HQ022771 |
| *T. neotropicale* | T51 | Peru | Hevea *brasiliensis* | / | FJ967825 |
| *[T. paratroviride](https://www.ncbi.nlm.nih.gov/nuccore/KJ665627.1)* | [S385 T](https://www.ncbi.nlm.nih.gov/nuccore/KJ665114.1) | Spain | / | KJ665321 | KJ665627 |
| *[T. paratroviride](https://www.ncbi.nlm.nih.gov/nuccore/MT454131.1)* | [PARC1012](https://www.ncbi.nlm.nih.gov/nuccore/MT454131.1) | / | / | MT454131 | MT454115 |
| *[T. paratroviride](https://www.ncbi.nlm.nih.gov/nuccore/MT822038.1)* | [H3-R-102](https://www.ncbi.nlm.nih.gov/nuccore/MT822038.1) | Mexico | *Coffea arabica* var. Bourbon | MT822038 | MT821993 |
| *[T. paratroviride](https://www.ncbi.nlm.nih.gov/nuccore/MT822038.1)* | [S489](https://www.ncbi.nlm.nih.gov/nuccore/MF185938.1) | / | / | KJ665322 | KJ665628 |
| ***[T. paratroviride](https://www.ncbi.nlm.nih.gov/nuccore/MT822038.1)*** | **T17** | **China** | ***G. sichuanense*** | **OR291402** | **OR291383** |
| ***[T. paratroviride](https://www.ncbi.nlm.nih.gov/nuccore/MT822038.1)*** | **T18** | **China** | ***G. sichuanense*** | **OR291403** | **OR291384** |
| ***[T. paratroviride](https://www.ncbi.nlm.nih.gov/nuccore/MT822038.1)*** | **T47** | **China** | ***G. sichuanense*** | **OR291417** | **OR291398** |
| *T. parestonicum* | CBS 120636 T | [Austria](https://wi.knaw.nl/page/fungal_display/name/javascript:void(0)" \o "https://wi.knaw.nl/page/fungal_display/name/javascript:void(0)) | *Hymenochaete tabacina* | FJ860565 | FJ860667 |
| *T. parepimyces* | CBS 122768 | / | / | FJ860563 | FJ860665 |
| *T. parepimyces* | CBS 122769 T | Austria | Wood | FJ860562 | FJ860664 |
| *T. peberdyi* | CEN1387 | Brazil | soil | MK696781 | MK696619 |
| *T. peberdyi* | CEN1388 | Brazil | soil | MK696782 | MK696620 |
| *T. perviride* | HMAS 273786 | China | Wood | KX026962 | KX026954 |
| *T. pinicola* | SFC20130926-S233 T | South Korea | Root of *Pinus densiflora* | MH025993 | MH025981 |
| *T. pinicola* | SFC20130926-S014 | South Korea | *Pinus densiflora* | MH025991 | MH025978 |
| *T. pinicola* | SFC20130926-S111 | South Korea | *Pinus densiflora* | MH025992 | MH025980 |
| *T. pleuroti* | CBS 124387 T | Korea | *Pleurotus* substrate | HM142372 | HM142382 |
| *T. pleuroti* | CPK 2117 | Hungary | / | / | EU279975 |
| *T. pleuroticola* | CBS 124383 T | Korea | *Pleurotus* substrate | HM142371 | HM142381 |
| *T. pleuroticola* | GJS 95-81 | / | / | / | AF348102 |
| *T. pleuroticola* | TRS70 ET | / | / | KP009172 | KP008951 |
| *T. pleuroticola* | T1295 | Canada | / | / | EU279973 |
| *T. polypori* | HMAS 248855 T | China | Soil | KY687994 | KY688058 |
| *T. polypori* | HMAS 248861 | China | Soil | KY688000 | KY688059 |
| *T. polysporum* | S72 | Italy | / | / | KJ665685 |
| *T. priscilae* | CBS 131487 T | Spain | / | KJ665333 | KJ665691 |
| *T. priscilae* | S129 | Italy | / | KJ665332 | KJ665689 |
| *T. pseudodensum* | HMAS 248828 T | China | Soil | KY687967 | KY688023 |
| *T. pseudodensum* | HMAS 248829 | China | Soil | KY687968 | KY688024 |
| *T. pseudogelatinosum* | CNUN309 T | South Korea | Shiitake mushroom | HM920173 | HM920202 |
| *T. pseudogelatinosum* | TUFC60186 T | Japan | Shiitake mushroom | JQ797405 | JQ797397 |
| *T. purpureum* | HMAS 273787 T | China |  | KX026961 | KX026953 |
| *T. pyramidale* | CBS 135574 T | Italy | *Olea europaea* | KJ665334 | KJ665699 |
| *T. pyramidale* | S573 | Italy | / | / | KJ665698 |
| *T. rifaii* | DIS 355B T | Ecuador | *T. gileri* | / | FJ463324 |
| *T. rifaii* | DIS 337F | Theobroma cacao | *T. cacao* | FJ442720 | FJ463321 |
| *T. rufobrunneum* | HMAS 266614 T | China | Rotten wood | KF730010 | KF729989 |
| *T. rufobrunneum* | HMAS 252547 T | / | / | KF730007 | KF729992 |
| *T. rugulosum* | SFC20180301-001 T | South Korea | / | MH025986 | MH025984 |
| *T. rugulosum* | SFC20180301-002 | South Korea | / | MH025987 | MH025985 |
| *T. simmonsii* | G.J.S. 91-138 T | USA | Decaying wood bark | FJ442757 | AF443935 |
| *T. simmonsii* | S297 | Croatia | / | / | KJ665711 |
| *T. simmonsii* | S7 | / | / | KJ665337 | KJ665719 |
| *T. simmonsii* | G.J.S. 90-22 | / | / | AY391925 | AF443933 |
| *T. simmonsii* | G.J.S. 92-100 | USA | / | FJ442710 | AF443937 |
| *T. simplex* | HMAS 248842 T | China | Soil | KY687981 | KY688041 |
| *T. simplex* | HMAS 248860 | China | Soil | KY687999 | KY688042 |
| *T. solum* | HMAS 248847 | China | Soil | KY687986 | KY688049 |
| *T. solum* | HMAS 248848 T | China | Soil | KY687987 | KY688050 |
| *T. solum* | HMAS 248849 | China | Soil | KY687988 | KY688051 |
| *T. spirale* | DAOM 183974 | / | / | AF545553 | EU280049 |
| *T. spirale* | LESF107 | Brazil | *Atta sexdens rubropilosa* | KT278956 | KT279022 |
| *T. stramineum* | GJS 02-84 T | Sri Lanka | Decaying wood | AY391945 | AY737746 |
| *T. stramineum* | TAMA 0425 | Japan | / | AB856748 | AB856675 |
| *T. tawa* | GJS 97-174 T | Thailand | Decaying bark | / | FJ463313 |
| *T. tawa* | DAOM 232841 | Thailand | / | KJ842187 | EU279972 |
| *T. tawa* | G.J.S. 97-174 T | Thailand | / | AY391956 | AY392004 |
| *T. tenue* | HMAS 273785 | China | Wood | KX026960 | KX026952 |
| *T. tomentosum* | DAOM 171918 | / | / | / | AY605759 |
| *T. tomentosum* | DAOM 178713A T | Canada | wood | AF545557 | EU279969 |
| *T. tomentosum* | DAOM 234236 | Guatemala | / | / | EU279971 |
| *T. tomentosum* | CBS 120637 | / | / | FJ860532 | FJ860629 |
| *T. tomentosum* | S33 | Italy | / | KF134793 | KF134801 |
| *T. velutinum* | DAOM 230013 T | Nepal | forest soil | JN133569 | AY937415 |
| *T. velutinum* | HMAS 273865 | China | Soil | KX026965 | KX026957 |
| *T. vermifimicola* | CGMCC 3.19850 | China | Compost | MN605870 | MN605881 |
| *T. vermifimicola* | HMAS 248255 T | China | Compost | MN605871 | MN605882 |
| *T. virens* | DIS 162 | Costa Rica | *T. cacao* | FJ442696 | FJ463367 |
| *T. virens* | DIS 328A | Ecuador | *T. gileri* | FJ442738 | FJ463363 |
| ***T. virens*** | **T20** | **China** | ***G. Sichuanense*** | **OR291405** | **OR291386** |
| ***T. virens*** | **T21** | **China** | ***G. Sichuanense*** | **OR291406** | **OR291387** |
| *T. xixiacum* | CGMCC 3.19697 T | China | Soil | MN605874 | MN605885 |
| *T. xixiacum* | CGMCC 3.19698 | China | Soil | MN605875 | MN605886 |
| *T. zayuense* | HMAS 248835 T | China | Soil | KY687974 | KY688031 |
| *T. zayuense* | HMAS 248836 | China | Soil | KY687975 | KY688032 |
| *T. zelobreve* | CGMCC 3.19695 T | China | Mushroom | MN605872 | MN605883 |
| *T. zelobreve* | CGMCC 3.19696 | China | Mushroom | MN605873 | MN605884 |
| *T. zeloharzianum* | YMF 1.00268 T | China | Soil | MH158996 | MH183181 |

**
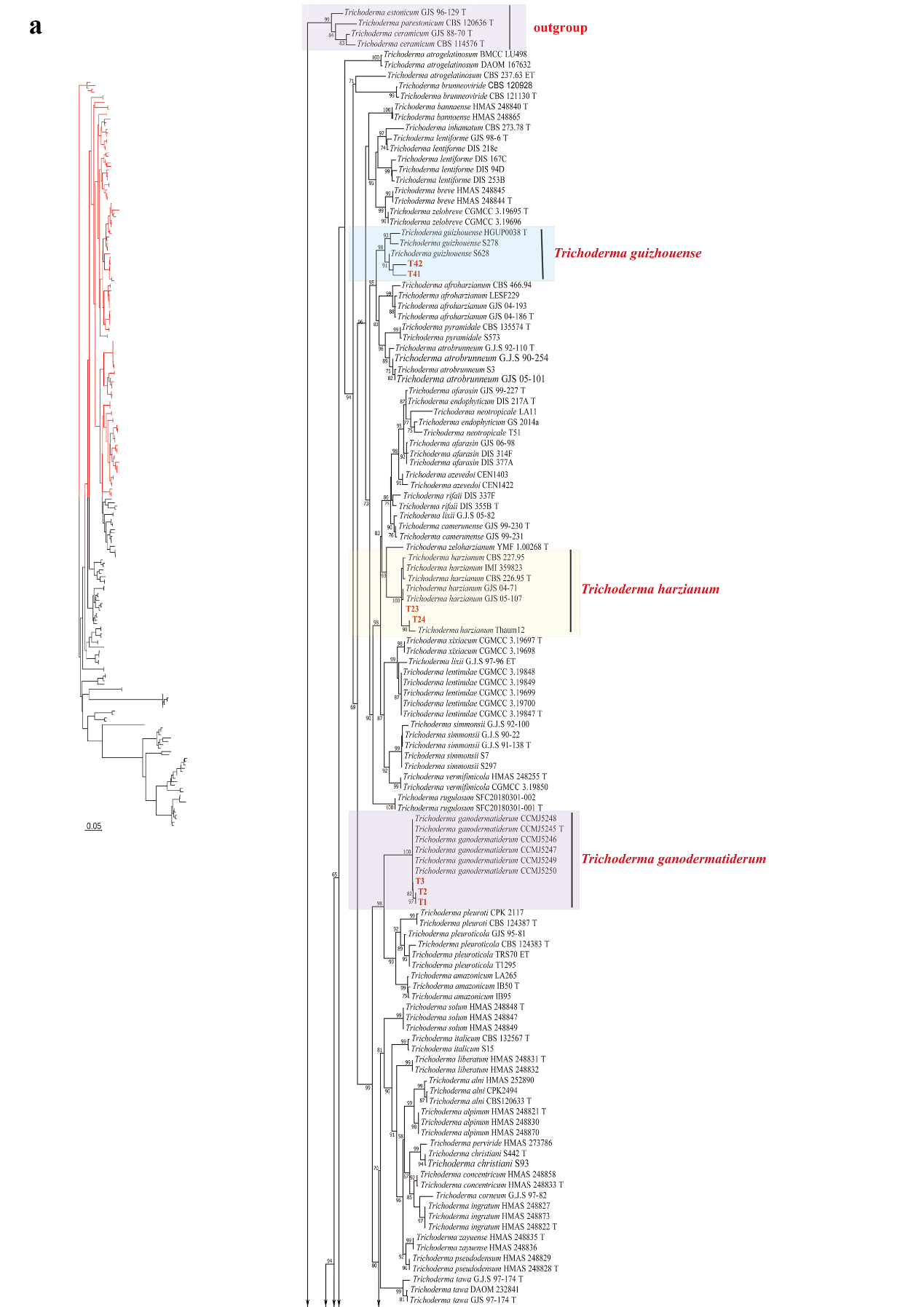
**

**
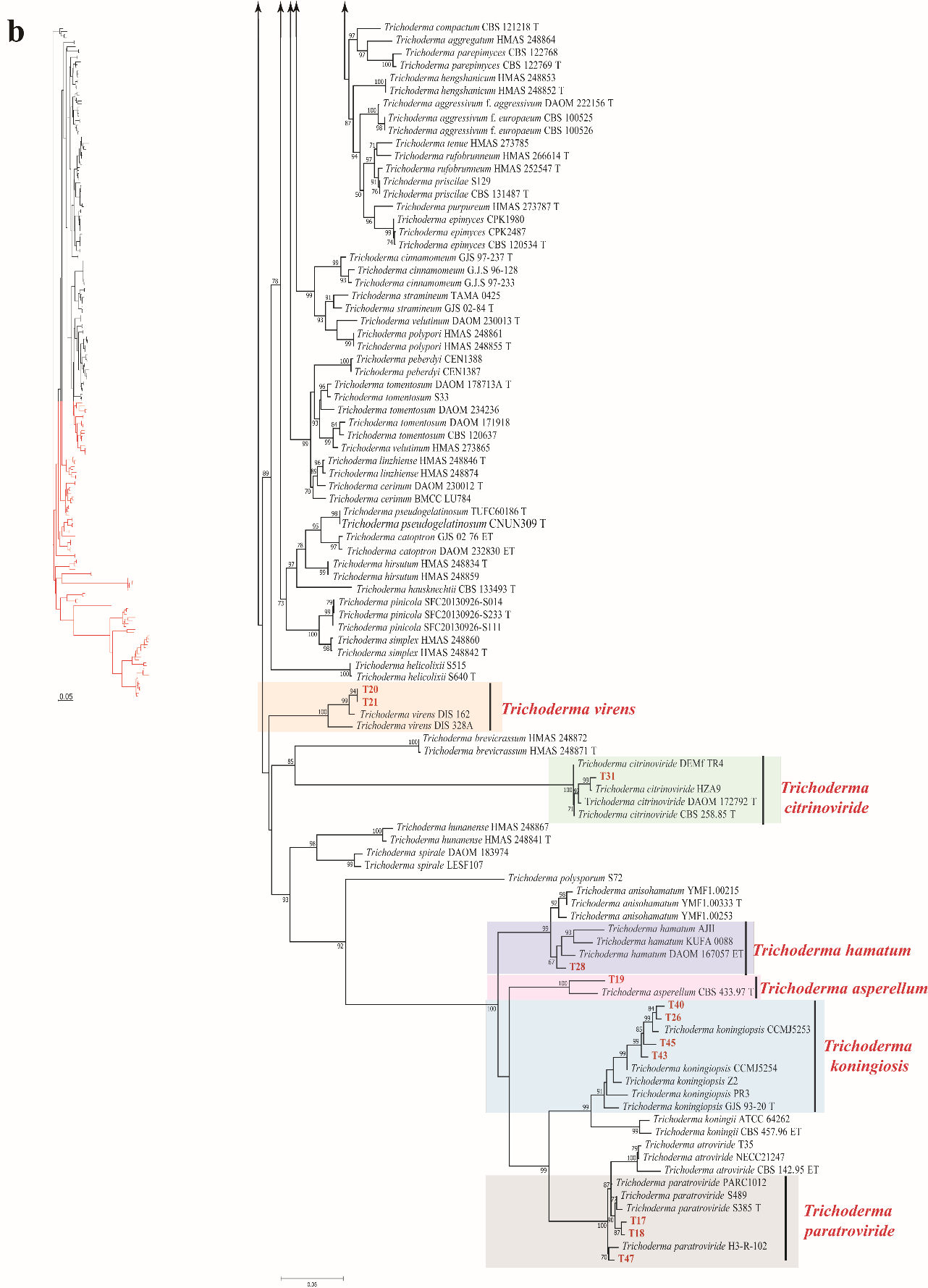
**

Figure S1. Phylogenetic tree obtained from maximum likelihood analysis based on the concatenated sequences of TEF1 and RPB2 genes. Support values at nodes representing PhyML bootstrap percent-ages with values ≥70 are shown above the branches.

**
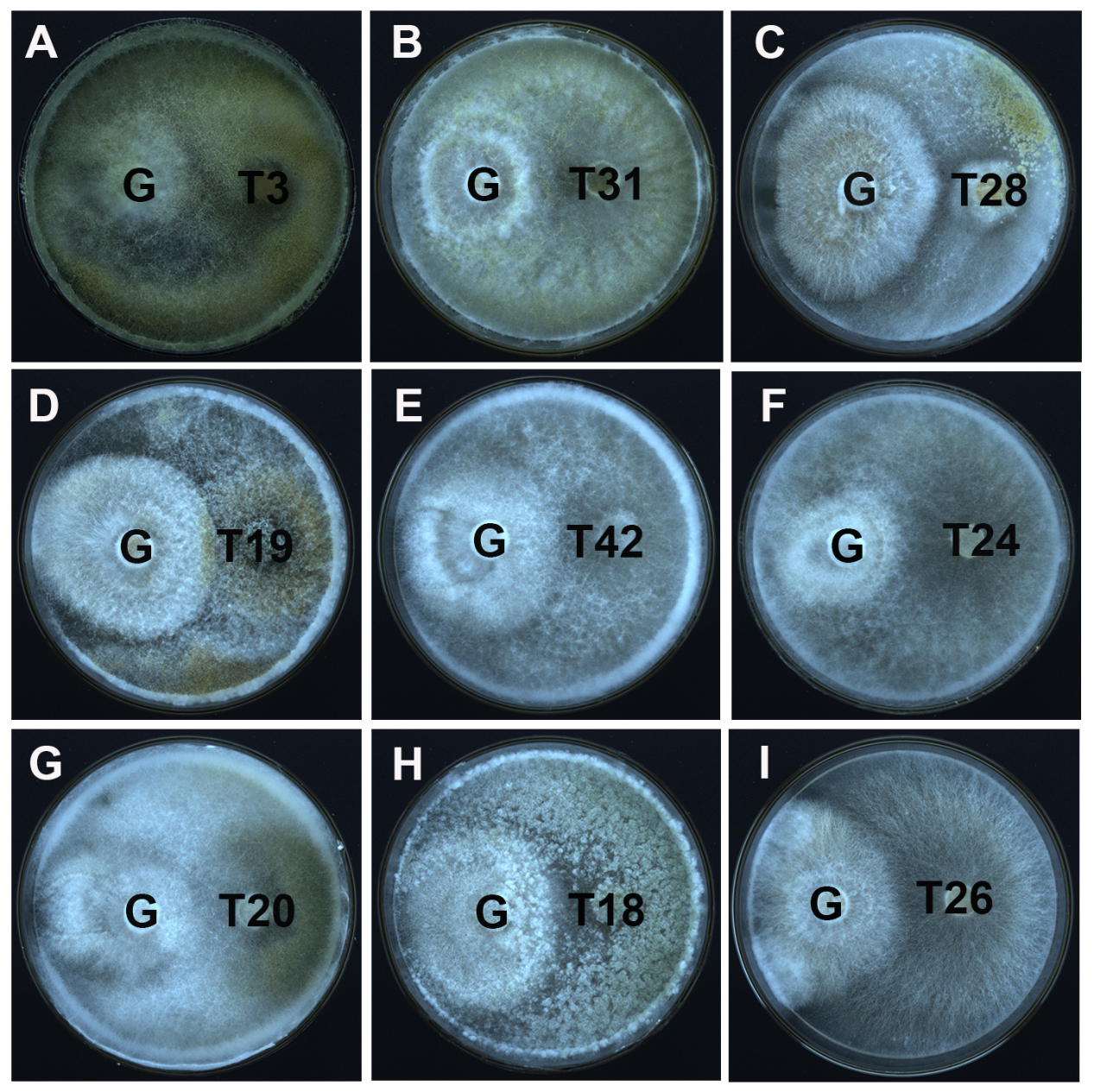
**

Fig. S2. Plate confrontation between *G. sichuanense* and *Trichoderma* (8d).

Note: T3: *T. ganodermatiderum*; T31: *T. citrinoviride*; T28: *T. hamatum*; T19: *T. asperellum*; T42: *T. guizhouense*; T24: *T. harzianum*; T20: *T. virens*; T18: *T. paratroviride*; T26: *T. koningiopsis.*


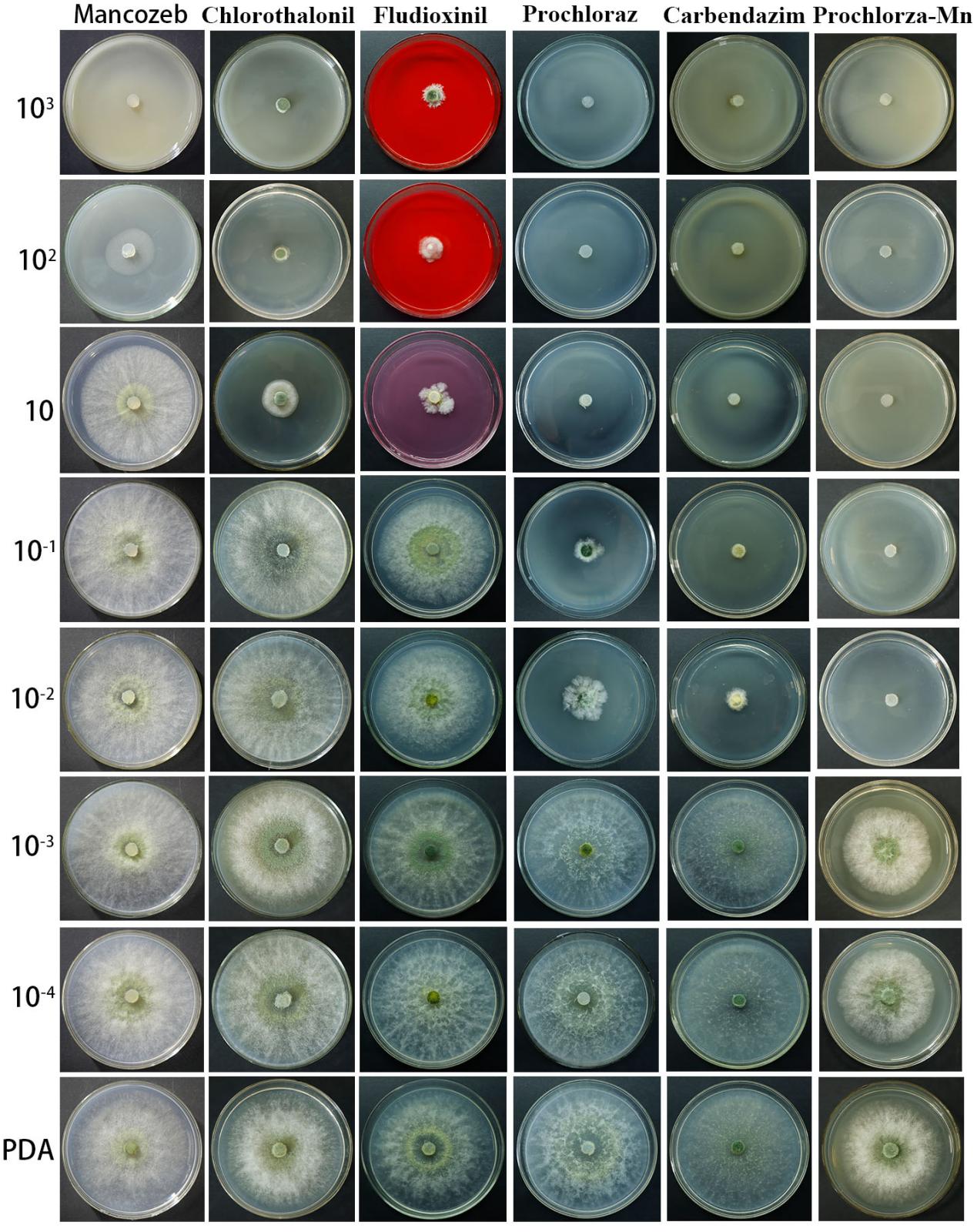


Fig. S3. The sensitivity determination of *T. ganodermatiderum* isolates to 6 fungicides.

Note: The units of different concentration gradients are μg/mL.


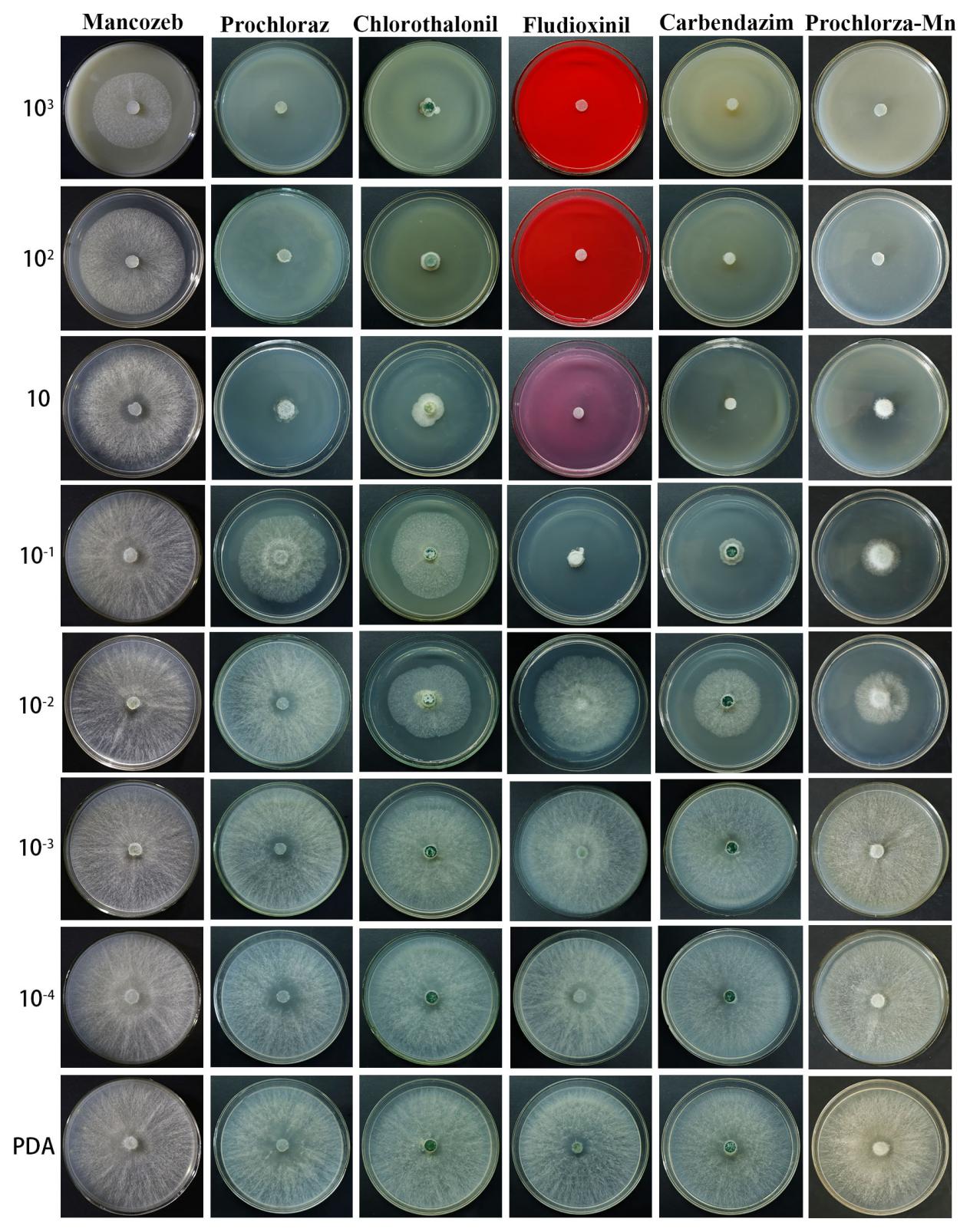


Fig. S4. The sensitivity determination of *T. paratroviride* isolates to 6 fungicides.

Note: The units of different concentration gradients are μg/mL.


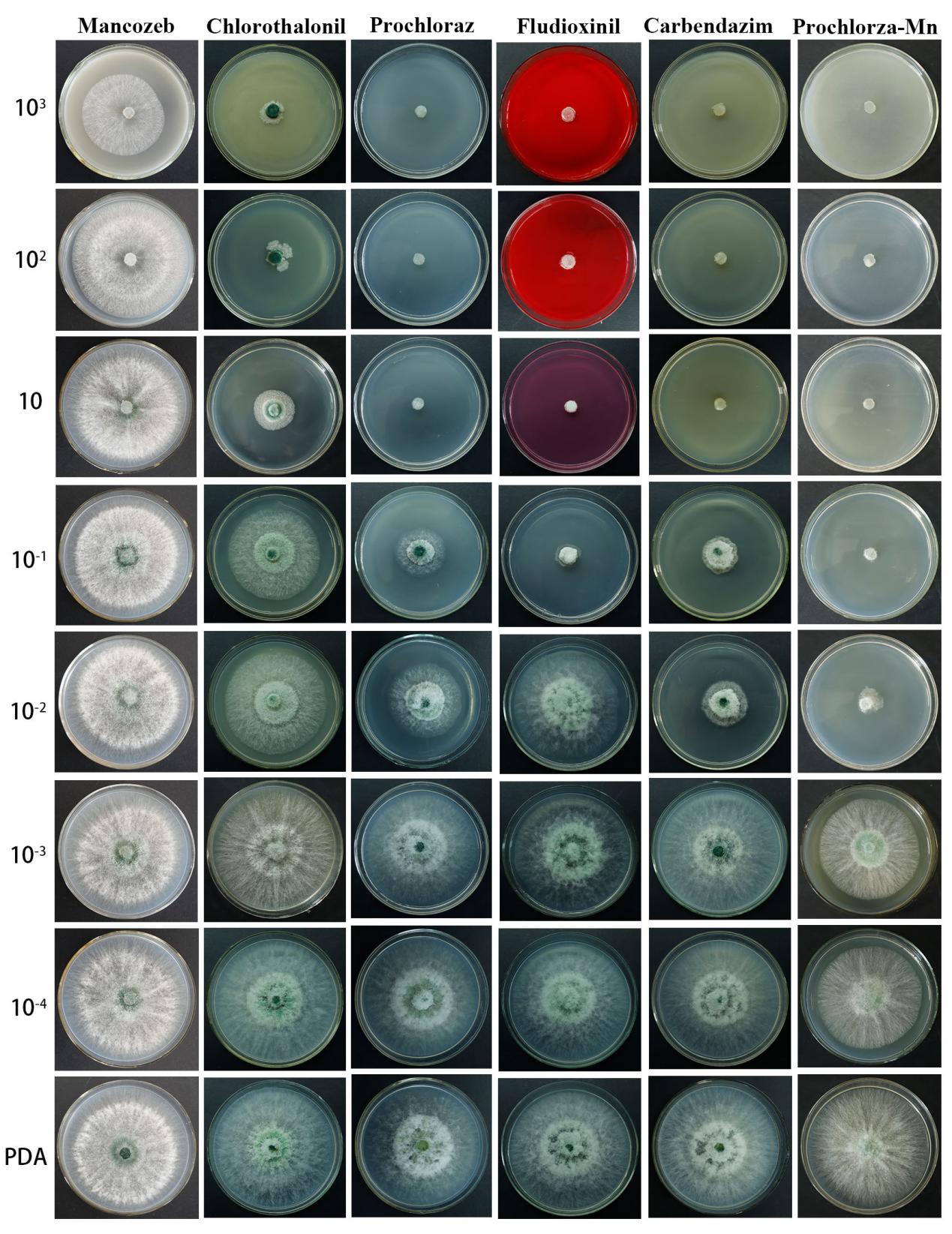


Fig. S5. The sensitivity determination of *T. asperellum* isolates to 6 fungicides.

Note: The units of different concentration gradients are μg/mL.

**
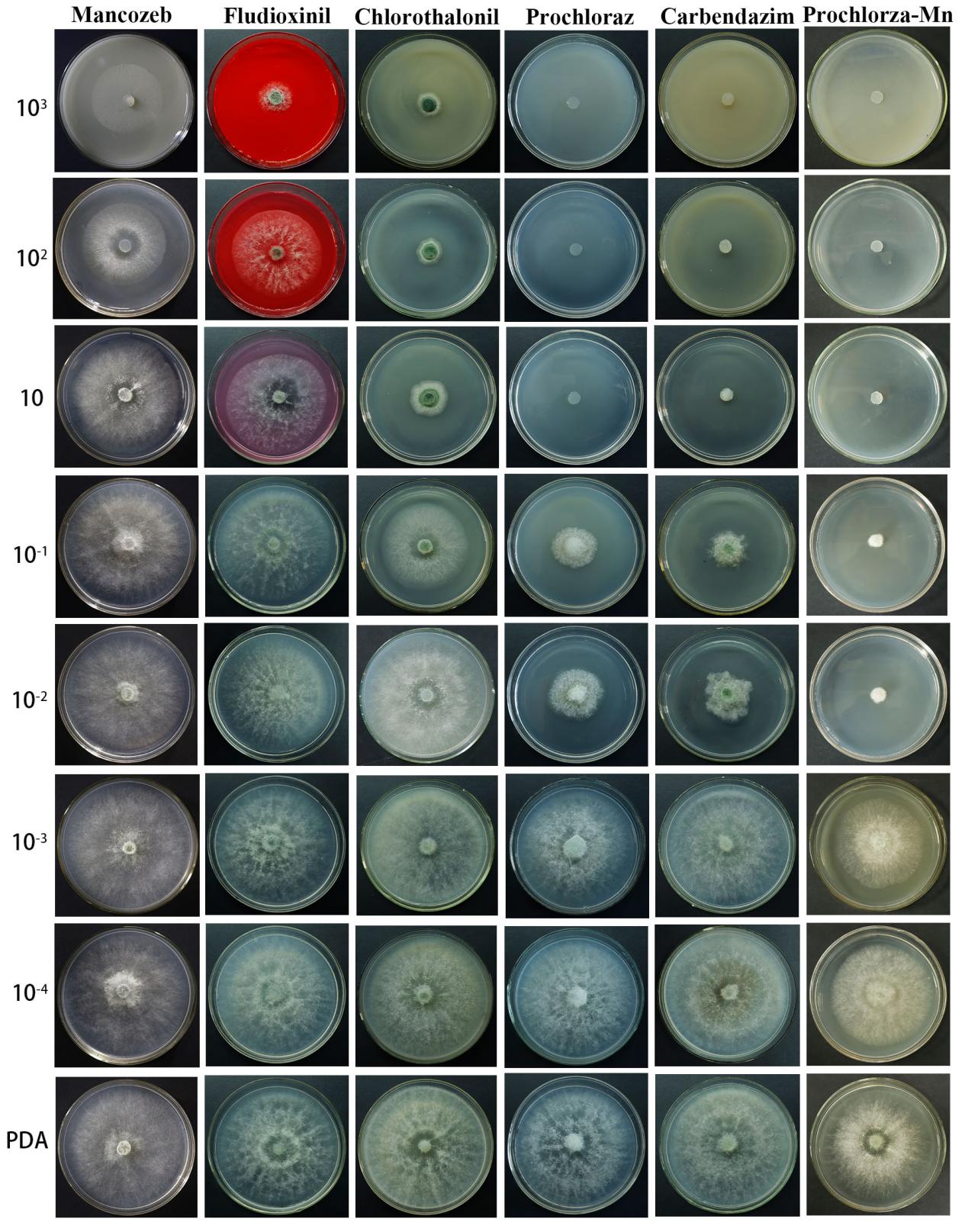
**

Fig. S6. The sensitivity determination of *T. virens* isolates to 6 fungicides.

Note: The units of different concentration gradients are μg/mL.


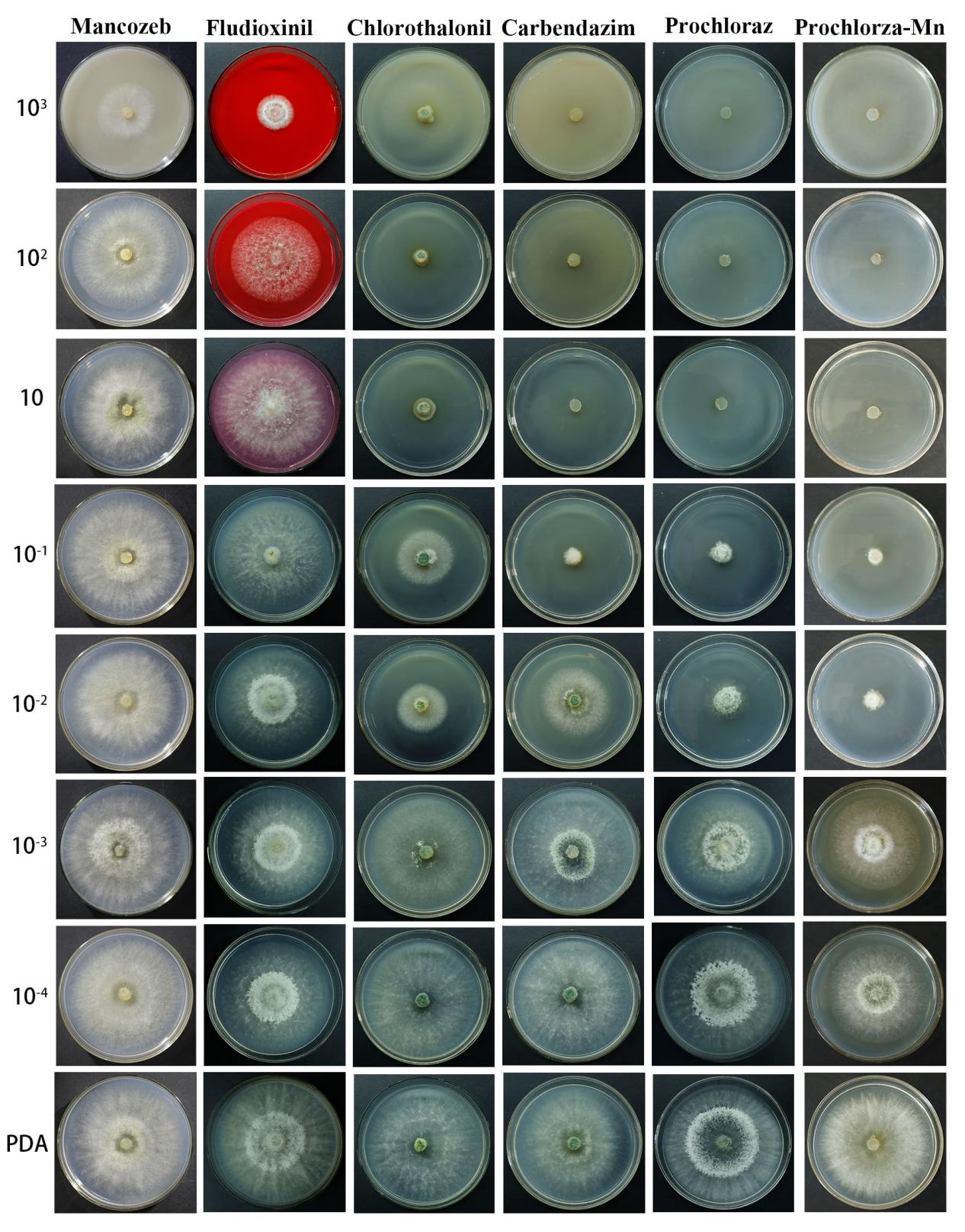


Fig. S7. The sensitivity determination of *T. harzianum* isolates to 6 fungicides.

Note: The units of different concentration gradients are μg/mL.

**
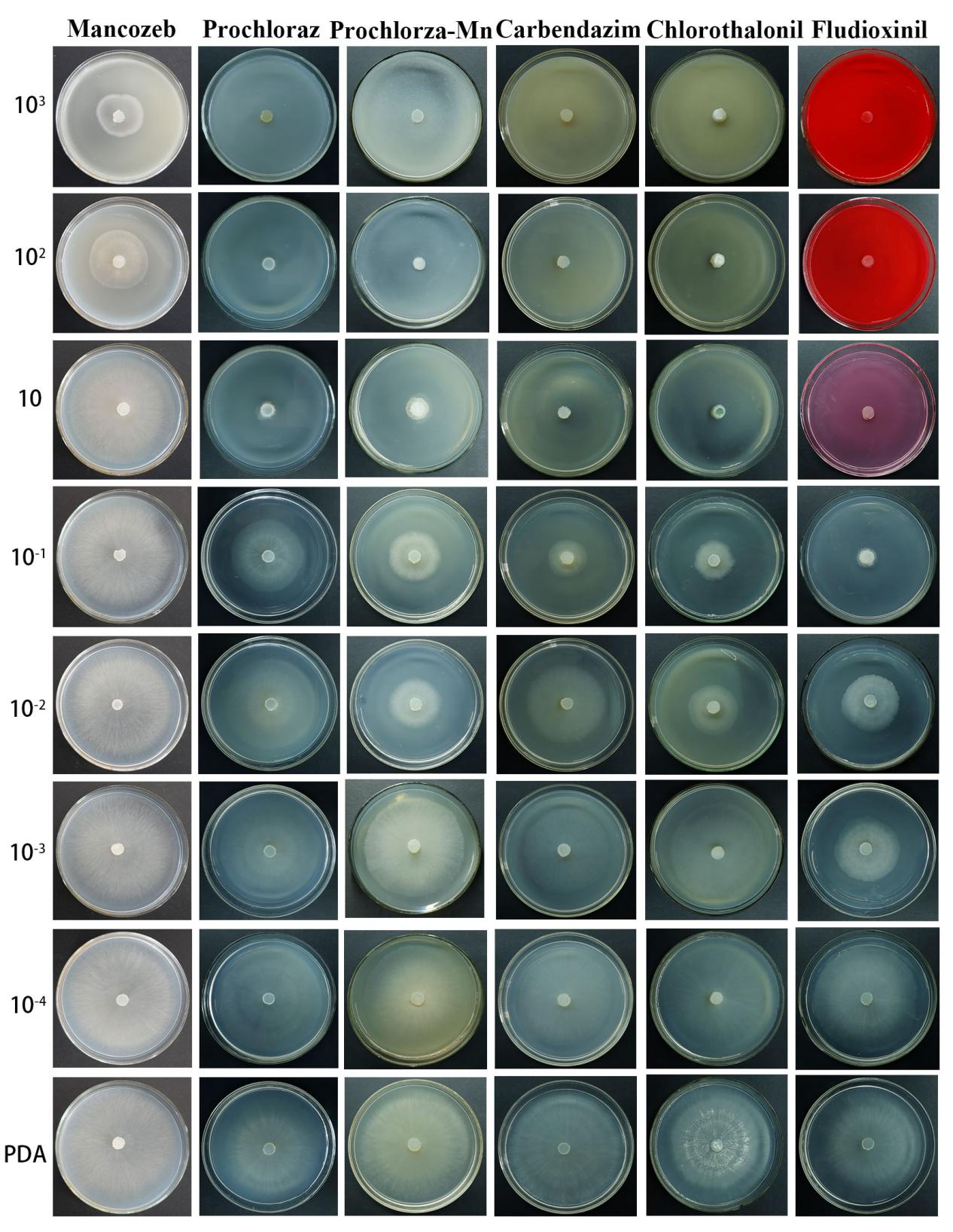
**

Fig. S8. The sensitivity determination of *T. koningiopsis* isolates to 6 fungicides.

Note: The units of different concentration gradients are μg/mL.

**
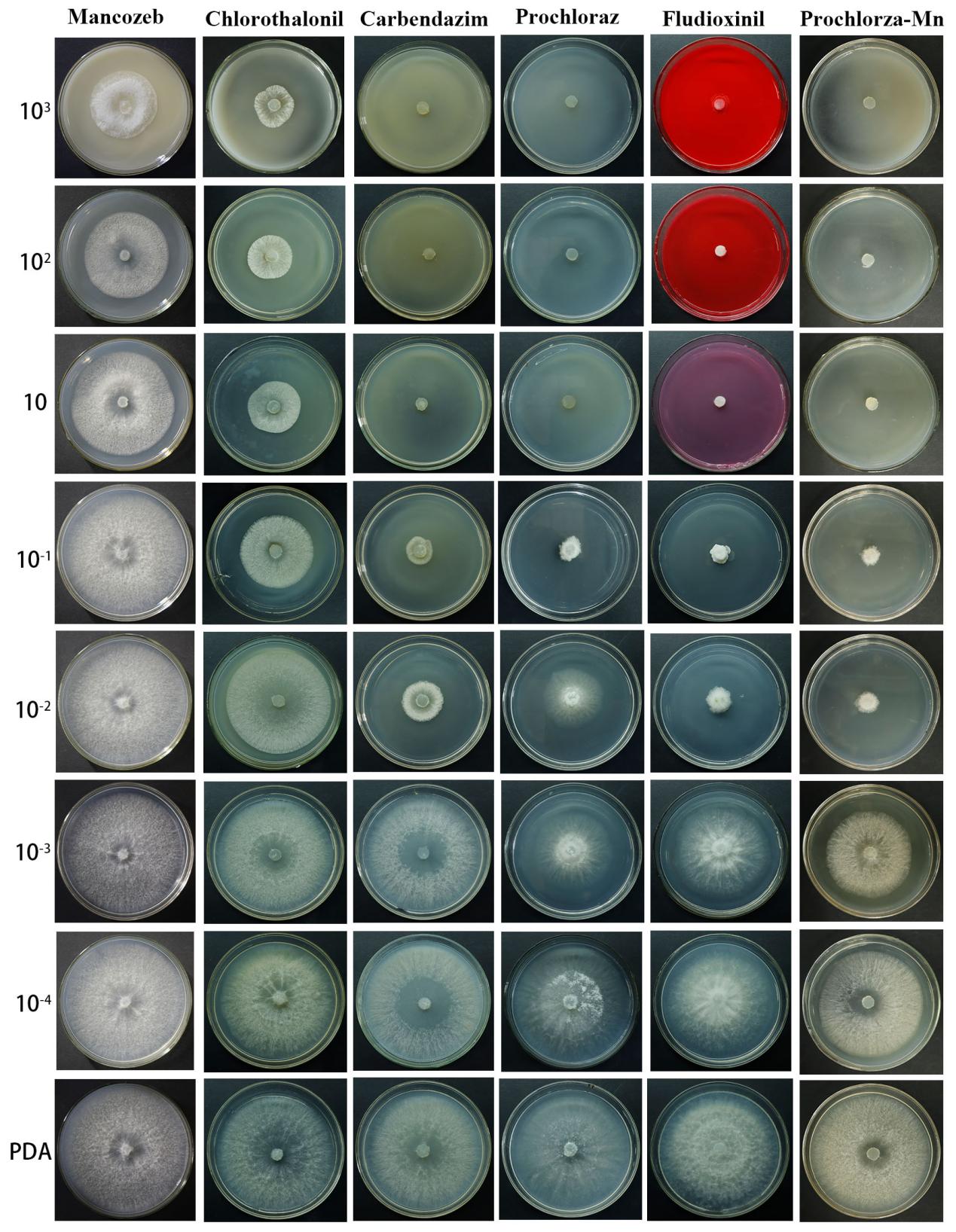
**

Fig. S9. The sensitivity determination of *T. hamatum* isolates to 6 fungicides.

Note: The units of different concentration gradients are μg/mL.

**
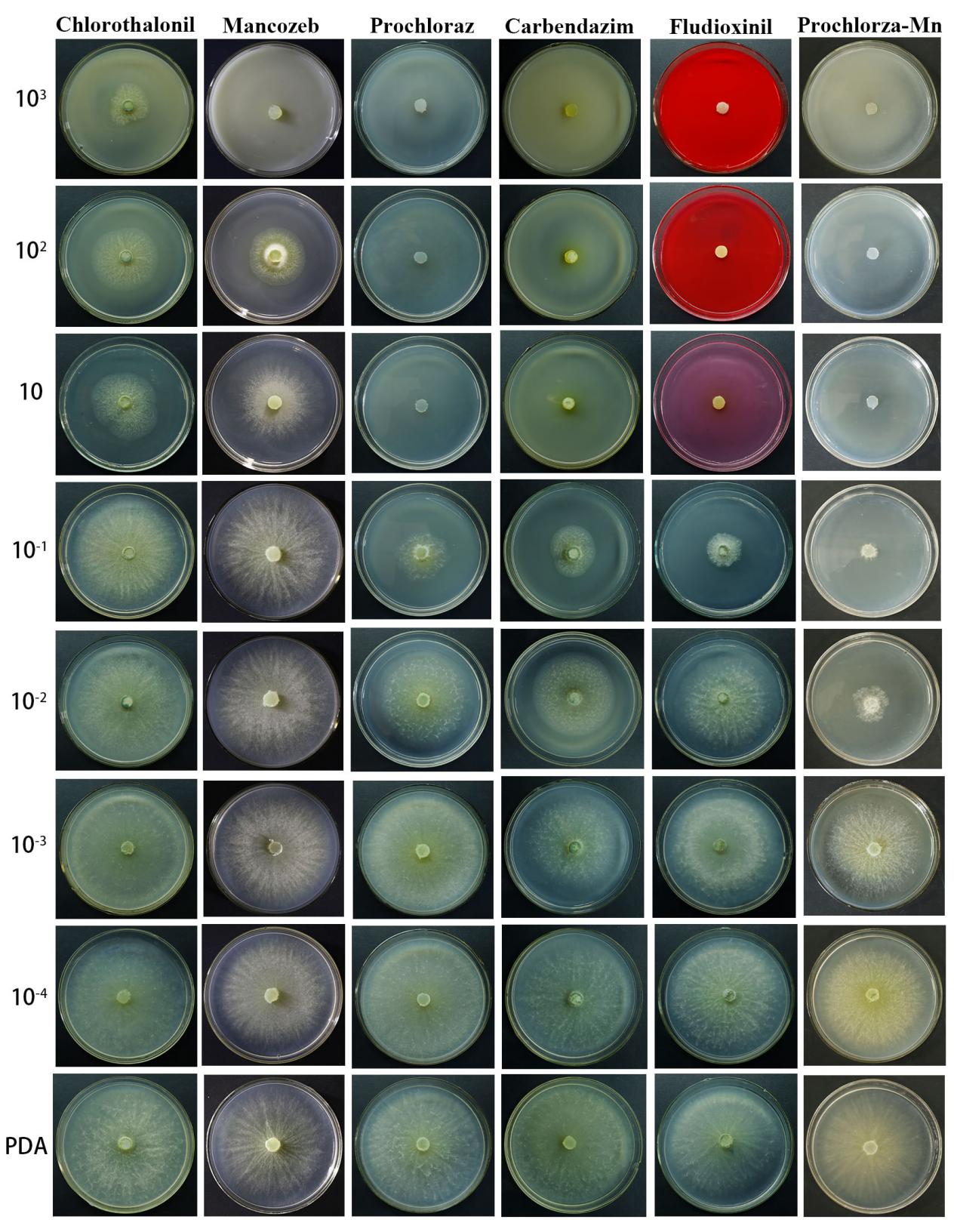
**

Fig. S10. The sensitivity determination of *T. citrinoviride* isolates to 6 fungicides.

Note: The units of different concentration gradients are μg/mL.


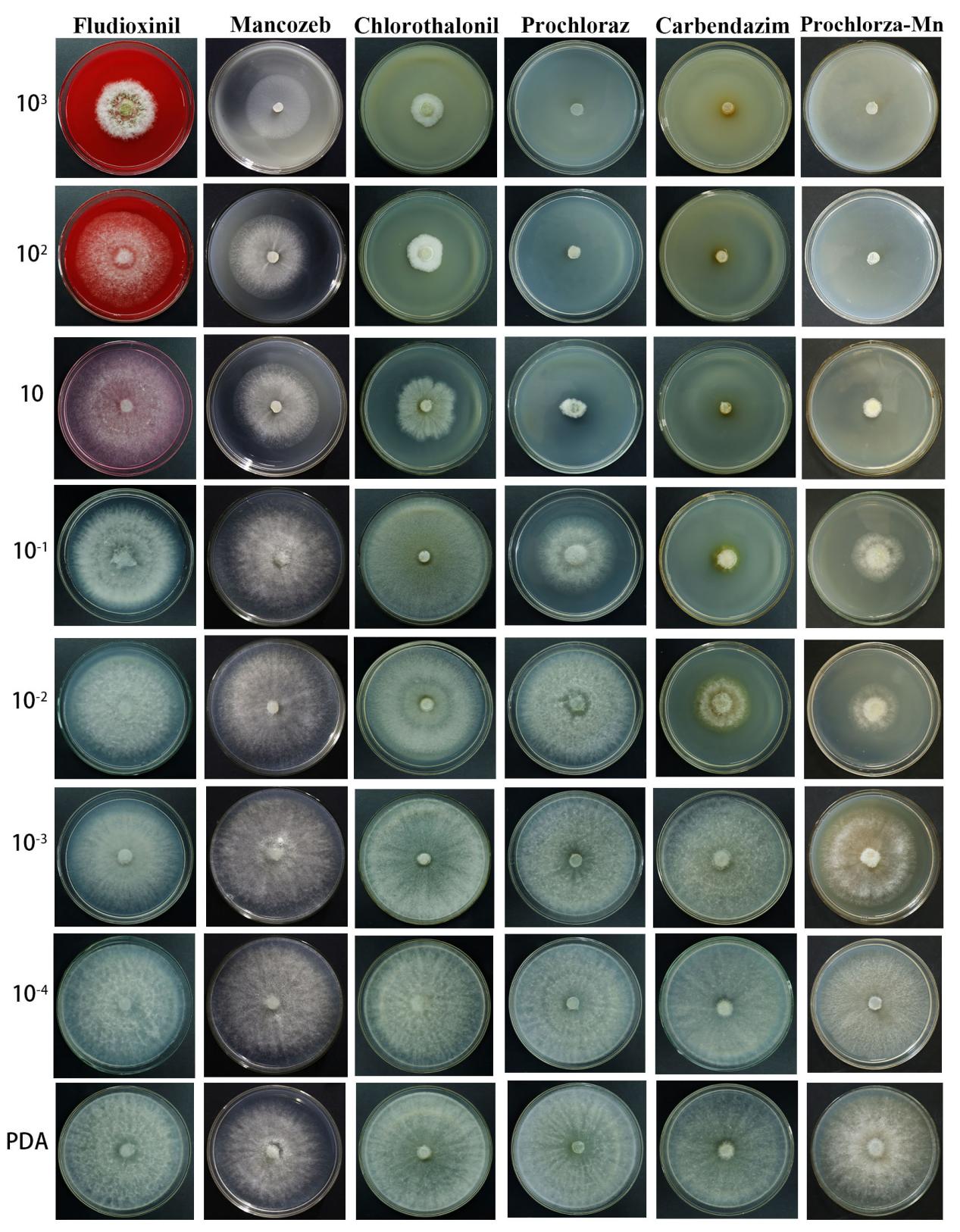


Fig. S11. The sensitivity determination of *T. guizhouense* isolates to 6 fungicides.

Note: The units of different concentration gradients are μg/mL.

**
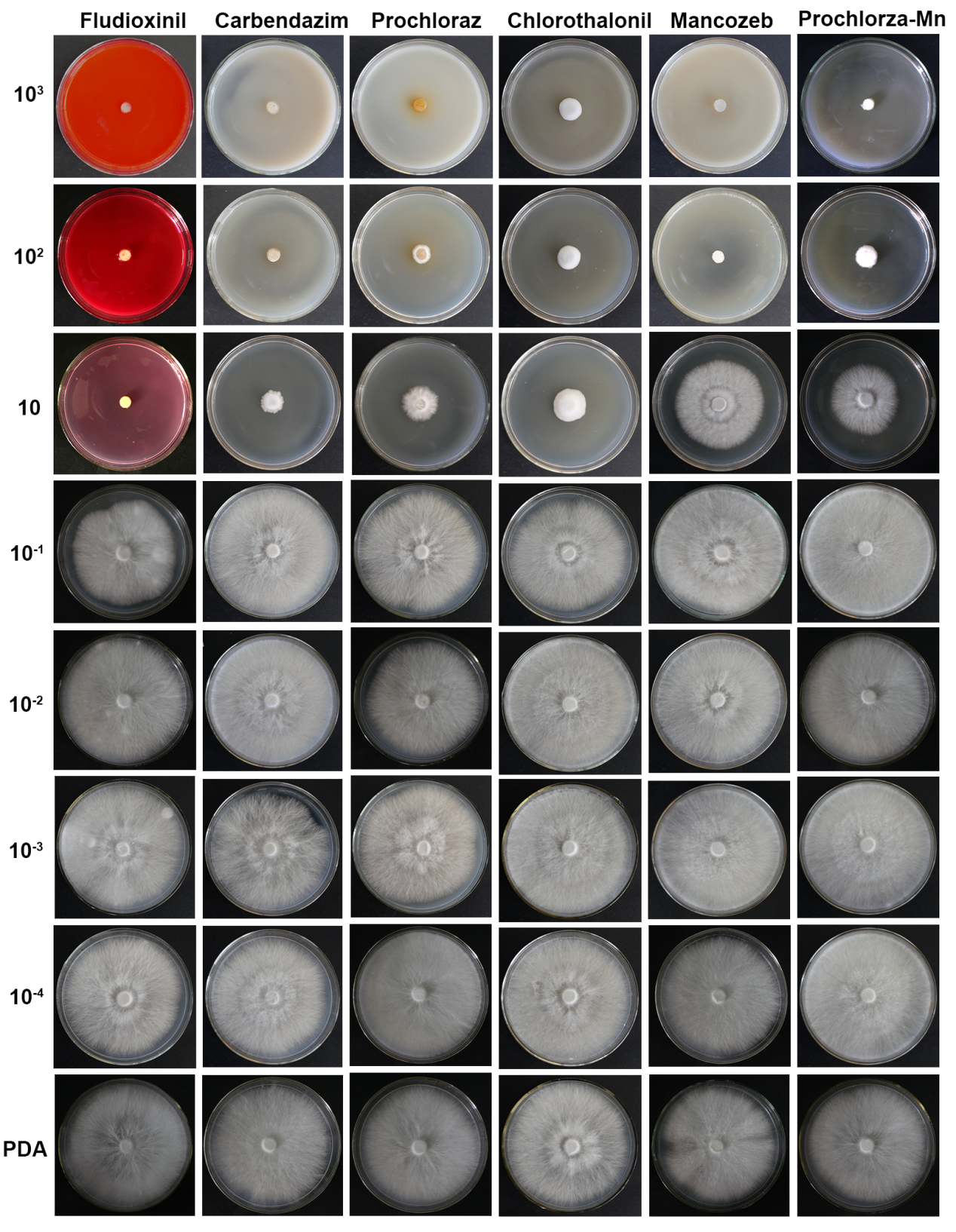
**

Fig. S12. The sensitivity determination of *G. sichuanense* isolates to 6 fungicides.

Note: The units of different concentration gradients are μg/mL.
